# Smart Manufacturing and Digitalization of Metrology: A Systematic Literature Review and a Research Agenda

**DOI:** 10.3390/s22166114

**Published:** 2022-08-16

**Authors:** Carlos Roberto H. Barbosa, Manuel C. Sousa, Maria Fatima L. Almeida, Rodrigo F. Calili

**Affiliations:** Postgraduate Programme in Metrology, Pontifical Catholic University of Rio de Janeiro, Rio de Janeiro 22453-900, Brazil

**Keywords:** smart manufacturing, digitalization of metrology, metrological services, systematic literature review

## Abstract

Smart manufacturing comprises fully integrated manufacturing systems that respond in real time to meet the changing demands and conditions in industrial activities, supply networks and customer needs. A smart manufacturing environment will face new challenges, including those concerning metrological issues, i.e., analysis of large quantities of data; communication systems for digitalization; measurement standards for automated process control; digital transformation of metrological services; and simulations and virtual measurement processes for the automatic assessment of measured data. Based on the assumption that the interplay between smart manufacturing and digitalization of metrology is an emerging research field, this paper aims to present a systematic literature review (SLR) based on a bibliographic data collection of 160 scientific articles retrieved from the Web of Science and Scopus databases over the 2016–2022 time frame. The findings presented in this review and recommendations for building a research agenda can help policy makers, researchers and practitioners by providing directions for the evolution of digital metrology and its role in the digitalization of the economy and society.

## 1. Introduction

The effective performance of smart manufacturing systems is rooted in their capacity and readiness to restructure and reconfigure industrial operations and supply networks to meet customer needs. Accordingly, measurement science problems have been considered critical for the coming smart manufacturing revolution [1,2,3,4,5]. Smart manufacturing comprises fully integrated manufacturing systems that respond in real time to meet the changing demands and conditions in industrial activities, supply networks and customer needs [4]. These systems integrate the manufacturing assets of today and tomorrow with sensors, computing platforms, communication technology, control, simulation, data-intensive modeling and predictive engineering [5]. A smart manufacturing environment will face measurement challenges and must manage uncertainty and unusual circumstances toward continuous improvement and evolution. So, the digital transformation of metrology is absolutely essential to achieve a digital quality infrastructure (DQI) for conformity assessment, product standardization, market surveillance and innovation in the most diverse smart manufacturing contexts. Furthermore, the digitalization of metrology can accelerate the measurement of products to reach their markets and shorten the time lags of approval processes.

Because the current revision of the metrological function is critical in several industrial applications and services, maintaining the global metrology infrastructure demands active collaboration. The global infrastructure is supported by the International Bureau of Weights and Measures (BIPM) and the National Metrology Institutes (NMIs), which are responsible for implementing and maintaining the national measurement standards. Moreover, the collaboration at the international level follows specific standards and agreements, e.g., the International Committee for Weights and Measures (CIPM) Mutual Recognition Agreement (MRA). Finally, to verify that the testing and calibration laboratories and metrological service providers fulfill all relevant regulations and standards (e.g., the ISO/IEC 17025 standard establishes general requirements for the competence of testing and calibration laboratories), the National Accreditation Bodies (NABs) supervise accredited testing and calibration laboratories and metrological service providers.

In the course of the digitalization of metrology, several initiatives have been conducted at the national, regional and international levels that could significantly impact the revision of the metrological function to face measurement challenges in the context of smart manufacturing operations [6,7,8,9,10,11,12,13,14,15,16].

The National Institute of Standards and Technology (NIST) in the United States of America (USA) has been building the metrological basis for implementing high-performance communication channels (5G) and creating regulatory and administrative foundations for the fields of cloud computing, big data, IT security and machine learning [6]. In Europe, the National Physical Laboratory (NPL) of the United Kingdom (UK) has been developing the data science field as well as 5G networks and digitalization-related research to face measurement challenges concerning the digitalization of industry [7]. In Germany, the Physikalisch-Technische Bundesanstalt (PTB) formulated a digitalization strategy in 2017 that has been implemented through several structuring projects around four focal points [8], as described later in this manuscript.

Similar initiatives are currently observed all over the world, as follows: (i) the National Conference of Standards Laboratories (NCSLI) initiative on Measurement Information Infrastructure (MII) in 2015 [9]; (ii) the Digital-SI Task Group of the International Committee for Weights and Measures (CIPM) in 2019 [10]; (iii) the European Metrology Cloud, a initiative to promote a pan-European coordinated IT infrastructure for legal metrology [11]; (iv) the Project SmartCom, focusing on communication and validation of smart data in IoT networks, which is a joint research project within the European Metrology Programme for Innovation and Research (EMPIR) [12]; (v) the Project GEMIMEG-II, addressing safe and robustly calibrated metrological systems for the digital transformation, aiming to develop a secure, consistent, legally compliant and legally binding end-to-end availability of information for the implementation of reliable, interlinked metrological systems in Germany [13]; (vi) the AnGeWaNt—*Arbeit an Geeichten Waagen fur Hybride Wiegeleistungen an Nutzfahrzeugen*—project is a joint research project in Germany addressing the automation of measuring instruments and their challenges for society. The six associates range from different areas, such as commercial partners in the weighing and construction industry, to a notified body in Germany, a local innovation hub (Zenitand GmbH) and the Institute for Applied Labor Science (IFAA) responsible for aspects within the social–economic and human factors [14]; (vii) the “Metrology for the Factory of the Future” (Met4FoF) initiative is another joint research project within the EMPIR initiative. It aims to establish a metrological framework for the complete lifecycle of measured data in industrial applications, i.e., from calibration capabilities for individual sensors with digital preprocessed output to uncertainty quantification associated with machine learning (ML) in industrial sensor networks [15]; and (viii) the Inter-NMI Experiment is an experimental blockchain network formed by National Metrology Institutes worldwide. It integrates servers provided by different NMIs and enables the implementation and testing of smart-contract-based applications related to metrology and conformity assessment. Research teams in each NMI can create their smart contracts and test them with support from other NMIs. This initiative started with a joint action of the German and Brazilian NMIs [16].

Of particular interest to this SLR is the strategic approach defined by PTB to the digital transformation of metrology in Germany, which emphasizes four focal points for research and development, namely: (i) digital transformation of metrological services; (ii) metrology in the analysis of large amounts of data; (iii) metrology of communication systems for digitalization; and (iv) metrology for simulations and virtual measuring instruments [8].

The first focal point—the digital transformation of metrological services—refers to the digital upgrading of the quality infrastructure and legal metrology by developing reference architectures and setting up a “metrology cloud” in the form of DQI for the harmonization and development of conformity assessment and market surveillance [8].

The second focal point refers to metrological analytical methods for handling, storage, security and reliability of a large amount of data, metrological traceability in the internet of things (IoT) and the use of cyber–physical systems, cloud computing, digital twins, artificial intelligence and machine learning in complex industrial contexts [8].

The third focus is on security and metrological validation of reliable, secure and efficient communication in complex industrial scenarios and implementing national DQIs worldwide. It encompasses the integration of IoT, cyber–physical systems and cloud computing for efficient communication and the traceability of complex high-frequency measurands for 5G networks [8].

Metrology for simulations and virtual measuring instruments—the fourth focal point—is concerned with the development of analytical methods and license procedures for interconnected and virtualized measuring systems, the simulation of complex measurements for automated process control, and virtual measurement processes [8].

In recent years, the research field of digital transformation of metrology has been increasingly growing. As a result of the growing interest in this subject, an escalating number of scientific and technical documents have been published from 2016 to 2022. By 2016, only 4 scientific documents had been published and indexed in the Web of Science database, while in 2021, the number of articles grew to 38. In the Scopus database, the figures are slightly higher, with 4 scientific documents published and indexed in 2016 and 63 in 2021.

Covering the time frame from 2016 to 2022 and focusing more specifically on previous studies that employed SLR or bibliometric approaches to provide a meta-analysis of what has been developed in this emergent research field, an initial literature search was conducted by accessing documents from the Web of Science and Scopus databases. This search yielded eight reviews [4,17,18,19,20,21,22,23], whose results are presented and discussed below.

Based on a bibliographic data collection of 396 documents, covering the time frame of 2010–2020, Sousa and Almeida [4] analyzed the interplay between metrology and nine emerging digital technologies, i.e., additive manufacturing; augmented reality; big data; cloud computing; cyber security; data integration; IIoT; network simulation; and autonomous robots. Using complementary bibliometric tools, they displayed a visual longitudinal science map over this time frame, highlighting digital technology trends and revealing their interactions and convergence of the nine digital technologies, considered as the Industry 4.0 pillars, which have been applied to metrology and vice versa.

Gadelrab and Abouhogail [17] reviewed 17 research papers published in the period from 2018 to 2020 covering the topics “digital transformation in metrology” and “digital calibration certificates” (DCCs). They identified that early research works were conceptual or theoretical, but in recent years, they evolved more toward implementation and experimentation. Another important observation refers to a tendency toward blockchain-based DCCs.

Varshney et al. [18] reviewed the leading advancements in sensor technology for digital metrological applications in different smart manufacturing contexts. They analyzed the impact of digital technologies on metrology along with implementing a “metrology cloud” and DQI. They concluded that to create a reliable national DQI and enhance the digital transformation in metrology worldwide, building “metrology clouds”, DCCs, and developing a measurement information infrastructure (MII), as proposed in Europe, will be absolute preconditions.

Dreyfus et al. [19] presented a SLR and an integrative conceptual framework aiming to discuss the virtual metrology (VM) use for product quality estimation in smart manufacturing. According to Ref. [19] (p. 742), “VM involves estimating a product’s quality directly from production process data without physically measuring it. This enables the product quality of each unit of production to be monitored in real-time while preserving the process efficiency”. The authors proposed a VM framework, which comprises the following elements: preprocessing, quality estimation, drift detection (DD), a sample decision system (SDS), the updatability feature and adaptability features, a multistage architecture, machine control and a fab-wide architecture. Each element was defined in terms of its functions and performance, emphasizing its potential for application in the most diverse manufacturing contexts.

Yang et al. [20] reviewed and synthesized the state-of-the-art research in interpolation and sampling design for surface measurement in varied manufacturing applications. They explored data-driven approaches that stem from statistics and machine learning and can potentially enable intelligent, cost-effective surface measurement and thus allow manufacturers to use high-resolution surface data for better decisions. Among these methods, the authors discussed how spatial and spatiotemporal interpolation techniques can draw inferences about unmeasured locations on a surface using the measurement of other locations, thus decreasing the measurement cost and time. Research gaps and future research directions are also identified and can serve as a fundamental guideline to industrial practitioners and researchers for future studies in these areas.

Nasir and Sassani [21] produced a critical review of the applications, opportunities and challenges associated with the data-driven approach applied to intelligent machining and tool monitoring focusing on deep learning (DL) methods. Among the opportunities of data-driven smart manufacturing approach applied to intelligent machining, the authors pointed out: automated feature engineering, handling big data and high-dimensional data, avoiding sensor redundancy, optimal sensor fusion and hybrid intelligent models. Moreover, they discussed the main data-driven challenges in smart manufacturing, e.g., those associated with the data size and nature and process uncertainty.

Catalucci et al. [22] reviewed the state of the art in optical metrology for digital manufacturing, discussing the main impacts of integrating optical coordinate and surface texture measurement technologies in smart manufacturing processes. Moreover, they presented the software and hardware that were applied to digital metrology and strategies for zero-defect manufacturing, together with an in-depth analysis of additive manufacturing applications. Finally, they identified challenges concerning bottlenecks in measurement speed and data processing; user-dependent constraints; harsh environments; geometric complexity, part size and surface texture; and uncertainty evaluation.

Ho et al. [23] conducted a SLR on augmented reality (AR) systems and their applications in smart manufacturing, covering the period from 2010 to 2021. Based on 200 documents selected from systematic searches in four scientific production databases, they found that there is an increasing concern in developing and implementing AR-assisted quality applications. Despite staying behind the assembly and maintenance sector in AR-based solutions, they highlighted three main categories of current AR-based solutions for the quality sector, namely AR-based apps as a virtual lean tool, AR-assisted metrology and AR-based solutions for in-line quality control.

Notwithstanding the contribution of these related works for describing the state of the art of this emerging research field, to the best of our knowledge, no systematic review or bibliometric study has been conducted that addresses all four focal points of the PTB’s strategic approach for the digitalization of metrology with implications for different sectors of the economy and public policies (Table 1).

Thus, the research questions addressed in this paper are:

- RQ1: What are the most significant challenges and opportunities of integrating metrology in a smart manufacturing environment, and which scientific efforts and initiatives have been developed to face them?
- RQ2: What are the latest trends for revising the metrological function to face measurement challenges in a smart manufacturing environment?
- RQ3: From the state-of-the-art review, which research directions can be identified to build a research agenda in this emerging field? Moreover, which research directions can be revealed?

To contribute to the advancement of knowledge on the interplay between smart manufacturing and digitalization of metrology as a research field, this paper aims to present a SLR and a research agenda based on a bibliographic data collection of 160 scientific articles retrieved from the Web of Science and Scopus databases over the 2016–2022 time frame. A final set of 70 scientific documents were selected and reviewed from the bibliographic data collection, following a three-stage approach proposed by Tranfield et al. [24] and Denyer and Tranfield [25]. Furthermore, the final set of documents were categorized according to the four focal points of the conceptual framework adopted in this review, which is based on the German NMI’s strategy for the digitalization of metrology [8].

The article is structured in five sections. Following the introduction, the second section describes the methodology adopted for searching relevant articles, refining the search and then making a final selection of the most relevant documents. Section 3 presents a descriptive analysis of the bibliographic data collection and the final set of documents to characterize the scientific production profile in this emerging research field. Section 4 presents and discusses the key findings regarding the in-depth literature analysis around the four focal points and 16 domains that integrate the mentioned conceptual framework.

Lastly, Section 5 synthesizes the concluding remarks and proposes a research agenda for those interested in advancing the research on the interplay between smart manufacturing and the digitalization of metrology.

## 2. Methodology

**Figure 1:**
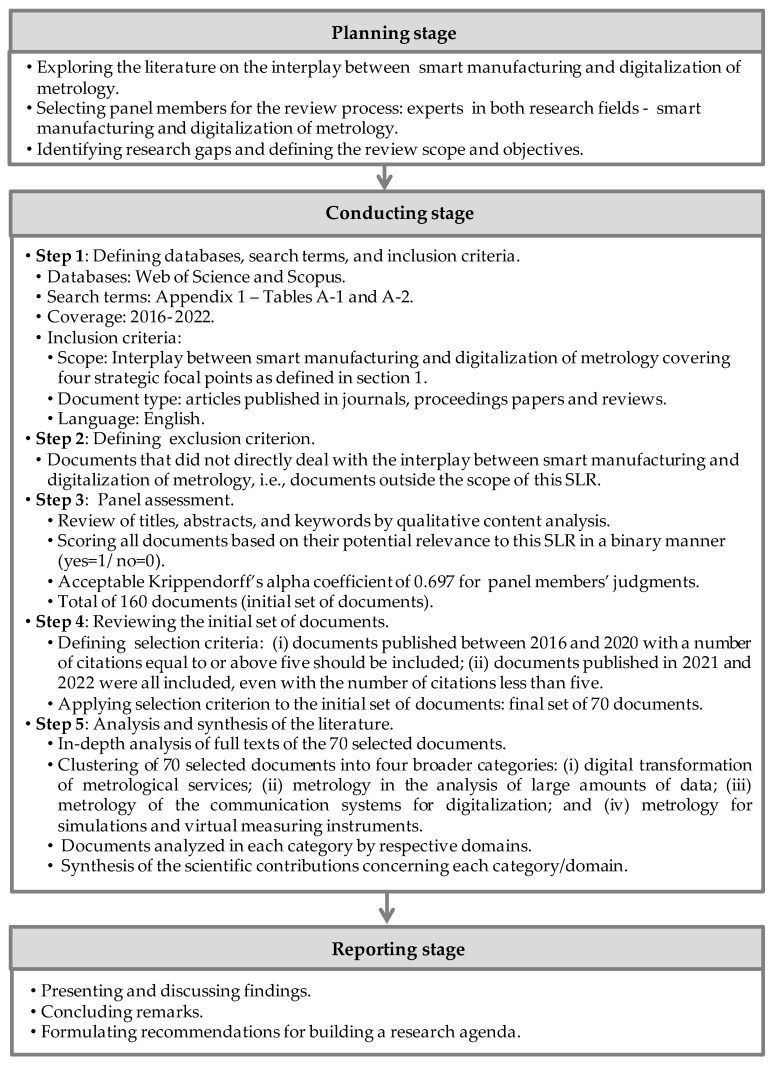
The literature review process’ schematic representation adapted from Ref. [26].

Figure 1 represents a schema of the SLR process following the three-stage approach proposed by Refs. [24,25]. To ensure that the findings are obtained in a reliable and valid manner, this review followed a three-stage approach, encompassing: (i) review planning; (ii) analysis of 160 documents and review of 70 selected ones; and (iii) reporting recommendations for building a research agenda that can help policy makers, researchers and practitioners by providing directions for the evolution of digital metrology and its role in the digitalization of the economy and society.

### 2.1. Planning Stage

The planning stage comprised three steps: (i) exploring the literature on the interplay between smart manufacturing and digitalization of metrology; (ii) selecting panel members for the review process; and (iii) identifying the research gaps and defining the review scope and objectives.

To integrate the review panel, three experts in both areas—smart manufacturing and metrology—were invited to identify and refine the study’s objectives and develop the review protocols. At this stage, an exploratory search on this research field in the Web of Science and Scopus databases covering the time frame 2016 to 2022 was conducted to identify previous studies that employed the SLR or bibliometric approaches. This search yielded eight related works [4,17,18,19,20,21,22,23], but none gave a comprehensive view of the interplay between smart manufacturing and digital metrology, addressing the four focal points of a conceptual framework based on the German NMI’s strategy for the digitalization of metrology. So, a research gap to be investigated was identified in the planning stage.

### 2.2. Conducting Stage

The conducting stage involved the systematic search on the Web of Science and Scopus databases covering the period from 2016 to 2022. The choice of the time frame and keywords was aligned with the evolution of scientific production in this research field, which began to emerge in 2016. The search histories in the Web of Science and Scopus databases are presented in Appendix A (Table A1 and Table A2, respectively).

After removing duplicates, the documents retrieved from the Web of Science and Scopus databases were organized on a Microsoft^®^ Office Excel worksheet (Microsoft Corporation, Washington, WA, USA), so that the three reviewers could analyze and assign the relevance scores independently. Hence, they analyzed the keywords, titles and abstracts of the downloaded documents to select those for this SLR based on the exclusion criterion previously defined, namely documents that did not directly deal with the interplay between smart manufacturing and digitalization of metrology and, therefore, were outside the scope of this SLR (Step 3—Panel assessment).

A systematic approach was followed as proposed by Refs. [24,25] throughout this stage to eliminate the risks of bias related to the SLR methodology’s inappropriate use. Moreover, the three reviewers’ participation and the proper definition of the exclusion criterion mitigated the risk of bias during the selection process. As a result of this process, 50 articles gained the highest score of 3 (cluster 1), 110 articles obtained a score of 2 (cluster 2), and 87 were excluded because they received a score of 0 or 1 (cluster 3). The panel members’ agreement met with an acceptable Krippendorff’s alpha of 0.697 [27].

Considering the relatively small number of documents obtained from the search strategies, the reviewers decided to include documents of the first and the second clusters for further analysis, totaling 160 documents. Backward citation search was not considered in this case, since the scientific production in this research field began to emerge in 2016, according to both databases’ search results.

Table 2 shows the bibliographic data collection of 160 documents, resulting from the preprocessing conducted with the support of Bibliometrix, an open-source R-package environment for bibliometric analysis developed by Aria and Cuccurullo [28].

In step 4 of the conducting stage, an objective selection criterion was clearly defined and understood by the reviewers, namely, that documents published between 2016 and 2020 with a number of citations equal to or above five should be included. Documents published in 2021 and 2022 were all included, even with the number of citations less than five. After applying the selection criterion to the initial set of 160 documents, 90 papers were excluded, resulting in a final set of 70 documents. The results of each step of the conducting stage of the SLR process are summarized in Appendix A (Table A3).

Based on an in-depth full-text analysis of the final set of documents, they were clustered into four broader categories corresponding to the mentioned strategic focal points, as follows: (i) digital transformation of metrological services (FP1); (ii) metrology in the analysis of large amounts of data (FP2); (iii) metrology of the communication systems for digitalization (FP3); and (iv) metrology for simulations and virtual measuring instruments (FP4). Finally, the subsets of documents were classified according to the domains of each category.

### 2.3. Reporting Stage

In the final stage of the review process, descriptive statistics and qualitative content analysis of the 70 selected documents were reported and discussed, and recommendations for building a research agenda were also outlined.

## 3. Descriptive Analysis

The scientific production profile in this research field covering the bibliographic data collection encompasses: (i) the annual evolution of scientific production comprising 160 documents (bibliographic data collection) and covering the time frame 2016–2022; and (ii) the classification of the final set of 70 documents into four broader categories (Table 3).

### 3.1. Evolution of Scientific Production in the Period from 2016 to 2022

**Figure 2:**
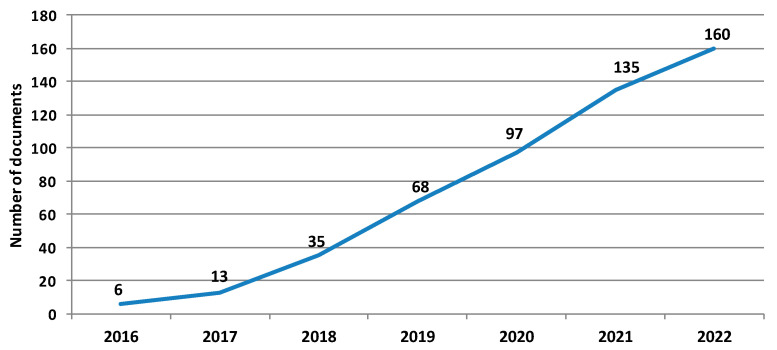
Evolution of the scientific production covering the bibliographic data collection: cumulative data from 2016 to 2022.

Figure 2 shows the growth of scientific production on the interplay between smart manufacturing and digitalization of metrology, covering the bibliographic data collection and the period from 2016 to 2022 (cumulative data).

Next, the types of publications were also investigated, and it was found that 54.4% (87 documents) of the 160 publications were from academic journals. In comparison, 46.3% (74 documents) of the publications consisted of conference papers, which was expected, as studies addressing the digitalization of metrology are relatively new and have mostly been presented in congresses and workshops. This result, together with the growing trend of publications in this time frame, points out that the digitalization of metrology and innovative digital applications in this area have increased and confirms the growing research interest and scientific production in recent years.

### 3.2. Clustering of the Selected Documents by Category

Table 3 shows the distribution of the final set of documents within the four categories mentioned in Section 2.2. In some cases, the articles showed the potential to fall into several categories. Nevertheless, an attempt was made to select the best category according to each article’s leading issues and metrological aspects. All the papers identified in each category are presented in separate tables in chronological order.

As seen in Table 3, most of the 70 documents are concentrated in two categories, namely “Digital transformation of metrological services” (24 documents) and “Metrology in the analysis of large amounts of data” (23 documents). The remaining categories, “Metrology of the communication systems for digitalization” and “Metrology for simulations and virtual measuring instruments”, with 12 and 11 documents, respectively, may indicate the current development stage of research in this research field, which opens up opportunities for future scientific works.

## 4. In-Depth Analyses of the Literature: Results and Discussion

This section presents and discusses the key findings regarding the in-depth literature analysis around the four focal points of the digital transformation of metrology in smart manufacturing environments.

### 4.1. Digital Transformation of Metrological Services

The digital transformation of metrological services creates additional value by extending features of services that would not have been possible previously, going beyond the update of the services by employing digitalization and the integration of existing metrological competencies of public and private entities. The transition toward digitalization of metrological services will significantly influence the quality infrastructure at the national and international levels and remove barriers to cross-border e-commerce and improve access to online content while increasing consumer protection.

Table 4 presents the reviewed scientific contributions related to this category [17,29,30,31,32,33,34,35,36,37,38,39,40,41,42,43,44,45,46,47,48,49,50,51] in chronological order and classified by five main domains of digital transformation of metrological services.

A qualitative content analysis of the full texts of the documents [17,29,30,31,32,33,34,35,36,37,38,39,40,41,42,43,44,45,46,47,48,49,50,51] enabled the identification of the five main domains concerning the digital transformation of metrological services, as presented in Table 4. They are: (i) digital calibration certificates; (ii) digital representation of metrological traceability; (iii) digital representation of physical quantities and units of measurement; (iv) digital representation of measurement error and uncertainty; and (v) digitalization in legal metrology and quality infrastructure. Accordingly, the documents were analyzed, and the results are synthesized below.

#### 4.1.1. Digital Calibration Certificates (D1.1)

Calibration results have been documented in calibration certificates, which have conventionally been printed as paper documents or PDF files. Consequently, interpreting the data in calibration management systems or other similar systems has required manual work, and calibration certificates have mostly not been available in a machine-readable format. For that reason, the first steps needed in the digitalization of metrological services have been defining and developing digital, machine-readable formats for presenting calibration information, i.e., DCCs [8,17,34,40].

DCCs use an internationally recognized extensible markup language (XML) format, which is flexible enough to become a worldwide standard in metrology [8]. The DCC defines an XML scheme, which is based on the minimum requirements for the machine-readable exchange of metrological data that are described in the D-SI metadata model developed within the European Metrology Programme for Innovation and Research (EMPIR) [12].

The DCCs comply with all international standards and guidelines required for such a document, including: (i) the SI [95]; (ii) the International Vocabulary of Metrology (VIM) [96]; (iii) the International Vocabulary of Legal Metrology (VILM) [97]; (iv) the Guide to the Expression of Uncertainty in Measurement (GUM) [98]; the Committee on Data for Science and Technology (CODATA) table review [99]; and the ISO/IEC 17025 standard [100].

Aligned with the ongoing digitalization of the metrology infrastructure and the above-mentioned normative references, some solutions that address the reliability validation of DCCs and their metrological traceability are highlighted in Refs. [33,34,41,48]. In particular, Brown et al. [34] presented an initial analysis concerning the requirements of a digital infrastructure to provide the required security and functionality for managing DCCs aiming to support the digital transformation of metrology.

As an alternative for data quality and reliability validation of these certificates, the solution developed by Mustapää et al. [33] refers to a conceptual model based on DCCs, D-SI and cryptographic digital identifiers. The data that enable validation and traceability can improve analytics, decision making and security in industrial applications.

In turn, Boschung et al. [41] proposed an approach for DCCs based on a PDF/A-3 solution that could be a stepping stone toward the digitalization of metrological services. They presented multiple applications of this approach by fulfilling the discussed minimum requirements and satisfying the needs of both customers and laboratories.

More recently, Mustapää et al. [48] developed a method for using digital formats for metrological data, including digital signatures and distributed ledger technology (DLT), alongside DCCs and D-SI to ensure integrity, authenticity and non-repudiation of measurement data and DCCs. The implementation of these technologies in industrial applications was demonstrated with a use case of data exchange in a smart overhead crane. The proposed system was tested and validated to provide security against data cyber attacks.

#### 4.1.2. Digital Representation of Metrological Traceability (D1.2)

As stated in the International Vocabulary of Metrology (VIM) [96], metrological traceability is “a property of a measurement result whereby the result can be related to a reference through a documented unbroken chain of calibrations, each contributing to the measurement uncertainty” [96] (p. 29).

Although metrological traceability is defined as an unbroken chain of calibrations to a national or international metrological standard, only the reference standard located immediately upstream in the calibration chain is reported on a calibration certificate. The GUM uses mathematical models to describe measurement processes and establishes a general approach to formulating such models and analyzing them to evaluate uncertainty. However, this Guide only briefly mentions the sequence of measurement stages needed to provide metrological traceability [36].

Hall and White [36] argue that measurement information needs to pass from one stage to the next along the traceability chain, and a minimum requirement for digital records of measured data is standard uncertainty reporting. The first finding from this study refers to the fact that conventional practice is almost exclusively related to reporting expanded uncertainty. So, as far as digital systems are concerned, a strategic effort is crucial to avoid replicating this practice. A second finding is that the reporting formats should encompass uncertainty components as much as possible, since digital systems can use this information in end-user applications.

Nowadays, blockchain technology constantly finds new applications for smart manufacturing, cyber–physical systems and digital metrological services. Its capability to ensure data integrity and authenticity makes this technology a reliable solution for the problem of digital representation of metrological traceability [41]. In this context, Softic et al. [42] emphasize that blockchain technology can be used in distributed digital measurements, sensors in smart devices and all measurement instruments that require calibration. Subsequently, digital representation of metrological traceability can be reinforced with blockchain-based infrastructure composed of accredited laboratories.

Another contribution came from Takatsuji et al. [32], who proposed a blockchain-based technology that allows the owner of a digital calibration certificate (DCC) to visualize the chain of calibrations to the primary standards. Currently, the owner of a calibration certificate lacks the data about the metrological traceability to an international standard, creating a space for falsification, since only the first higher standard is reported in the calibration certificate. As argued by Ref. [32], the blockchain-based system assures the authenticity and integrity of the DCCs and access control by using timestamps secured with hash functions in each block. However, the proposed system works effectively only when all the calibration laboratories in the traceability chain use it.

In metrology regulation contexts, implementing blockchain-based technologies must consider the technical aspects and the intrinsic legal framework. Based on the assumption that normative standards and legal requirements are crucial for building trust in metrology regulation contexts, Milicevic et al. [51] defined a trust model concept for IoT blockchain, starting from the IoT device level, for analyzing the possibility of implementing the WELMEC standard [101] using the blockchain and going through levels of metrology hierarchy up to the sole definition of the measurement unit. Although its applicability could be demonstrated by analyzing the WELMEC standard for determining software risk categories in a measuring device, the authors concluded that for practical applications, it would be necessary to analyze blockchain properties and applicability with a view to the standard requirements.

#### 4.1.3. Digital Representation of Physical Quantities and Units of Measurement (D1.3)

Digital representations of physical quantities and units of measurement are urgently needed to support the digital transformation of national and international quality infrastructures [37,48]. According to Chalk et al. [43], the SI redefinitions adopted in 2019 did not address the representation of SI units and did not extend to digital representations. In this regard, the International Committee for Weights and Measures (CIPM) has created a Task Group on the Digital SI in 2019 [10] aiming at: (i) developing and establishing an agreed worldwide, uniform, unambiguous, authoritative and dependable data exchange framework based on the SI described in the current SI Brochure [52]; (ii) proposing suitable actions toward making the SI Brochure machine readable; and (iii) coordinating this effort with all relevant stakeholders by exploring or establishing suitable liaisons.

Brown et al. [50] discussed the recent revision of the SI [52], reinforcing the need to provide a digital framework for the SI that will allow machine-readable measurement reporting, agreed metadata standards, understanding of measurement uncertainty in complex systems and validation of data analysis algorithms.

Digital representations of physical data should incorporate additional metrological information to complement that already captured in standard unit notations. However, difficulties in representing physical quantities have emerged in digital systems, which relate to how we think about quantities, dimensions and units. Addressing this concern, Hall and Kuster [37] outlined an interpretation of these concepts and their relations to address the representational obstacles. They proposed a layer of metrological information to enable familiar unit formats for users, so that systems can unambiguously exchange and process data. Accordingly, the layer should handle three independent data aspects: (i) the quantity; (ii) the measurement scale, scale type and conversion functions; and (iii) the semantics of numerical values.

#### 4.1.4. Digital Representation of Measurement Error and Uncertainty (D1.4)

Since the early 1990s, the GUM [55] has been influential in promoting uniform reporting formats for the representation of measurement error and uncertainty within national quality infrastructures (NQIs) and has been widely adopted in laboratories and organizations. In the course of the digitalization of the industry, NQIs have been preparing to digitalize their operations. So, sharing digital records will soon be imperative, and therefore, the principles enunciated in the GUM must be carefully reconsidered from the perspective of digital implementation.

With this concern, Smith et al. [47] discuss the storage of uncertainty information within DCCs. They argue that DCCs provide two key benefits that immediately aid the reporting and use of a complete set of Monte Carlo results. They are: (i) the information being provided in a fully machine-readable form; (ii) the potential to include a much larger amount of data than in a paper-based or electronic certificate. DCCs, therefore, provide the means to transfer uncertainty information that is encapsulated in a set of Monte Carlo samples. The authors present two examples to demonstrate how DCCs can allow a Monte Carlo calculation’s complete set of results to be reported. However, one should be aware of the circumstances under which the use of a Monte Carlo approach does not support the transferability of results.

Hall and White [36] argue that the representation of measurement uncertainty in digital records should not follow the current practices. Instead, they recommend that reporting formats include uncertainty components wherever possible because digital systems can use this information in end-user applications. In this regard, Hall and Koo [37] considered a future scenario in which digital reporting of measurement results should be ubiquitous, and DCCs contain information about uncertainty components in a measurement result. They developed a case study to look at the benefits of digitalization, using international measurement comparison linking. This approach provided a context in which correlations are essential, so they could evaluate the benefit of passing a digital record of contributions to uncertainty along a traceability chain.

#### 4.1.5. Digitalization in Legal Metrology and Quality Infrastructure (D1.5)

Legal metrology is defined in the International Vocabulary of Legal Metrology [54] (p. 16) “as the part of metrology relating to practice and process of applying statutory and regulatory structure and enforcement to metrology”. The main purpose of legal metrology is to establish and provide trust among all stakeholders, such as customers, manufacturers and users of measuring instruments.

In the context of digitalization in legal metrology, Peters et al. [30] highlighted applications of blockchain-based technologies for measuring instruments under legal control and identified promising areas, e.g., the complete automation of the legally supervised update mechanism by smart contracts. Nonetheless, they argued that blockchain-based technologies still need time and research efforts to be implemented in legal metrology. From the same perspective, Melo [47] presented the main concepts that bind legal metrology to blockchain-based technologies. According to Ref. [47], the demand for blockchain-based applications will increase over the coming years, and legal metrology can also take advantage of that. Moreover, these digital technologies will also require help from legal metrology to manage the physical assets correctly.

Melo et al. [31] discussed how blockchain-based technologies could be used to support distributed measuring systems (DMS), exploring concepts related to the integration of measuring instruments in DMS. As the main results of their research, a blockchain-based model was proposed based on the assumption that blockchain-based technologies can improve metrological assurance of measurement instruments by imposing restrictions against potential attacks while reducing technical efforts related to regulation and control activities. Moreover, a security analysis was developed to demonstrate that the proposed architectural model could improve measurement instruments’ security by constraining cyber attacks. However, despite its promising application, the authors drew attention to several challenges posed by blockchain-based technologies, such as a large amount of data, privacy and oracles’ authentication.

Aiming to foster the digital transformation in legal metrology in Europe, PTB initiated the development of a coordinated European DQI for innovative products and services, called the European Metrology Cloud (EMC) [11]. The EMC project encompasses 16 measuring instrument classes in a supranational context of European legislation. Its foundation lies in a trustworthy metrological core platform in each member state, designed to support and streamline regulatory processes by joining existing infrastructures and databases. This DQI will provide reference architectures, i.e., new measuring instruments and technology- and data-driven digital services for legal metrology [29,35,39,49]. Considered a national spinoff of the EMC, the AnGeWaNt project [14] focuses only on weighing instruments and aims to rationalize and improve the metrological processes for this class of instruments under legal control [35].

Keidel and Eichstädt [40] demonstrated how individual processing steps can be combined into a harmonious overall digital process by choosing a suitable IT infrastructure that supports these data and has suitable interfaces for modularization. The authors used the example of the digital transformation of metrological services at PTB and provided evidence of how this approach can be translated into an interoperable DQI. They argued that, for a successful and harmonized digital transformation of complex processes, it is essential to perform a complete analysis of the entire lifecycle of the required and processed data. Furthermore, these data should be as standardized, universally machine readable and internationally recognizable as possible concerning future extensions.

Grasso Toro and Lehmann [45] summarize the research and development (R + D) efforts made by two influential NMIs in Europe, i.e., the National Physical Laboratory (United Kingdom) and the PTB (Germany). Aiming to give a representative overview of the digitalization efforts of NMIs in Europe, they also present the digitalization strategy of the Federal Institute of Metrology (METAS) (Switzerland).

Finally, Garg et al. [44] analyzed and discussed the various aspects of the digital transformation in metrology in India, which shall be pivotal in establishing a metrology cloud and a national DQI in this country. Such transformation requires implementing the IoT-based digital framework, cloud computing, big data and digital twins. Moreover, the study presents the SWOT analysis of the digital transformation in metrology in India over the next few years.

Some of the topics covered in this item concerning digitalization in legal metrology and quality infrastructure will be discussed in Section 4.3—”Metrology of the Communication Systems for Digitalization”.

### 4.2. Metrology in the Analysis of Large Amounts of Data

Table 5 summarizes the contributions of 23 selected documents addressing the second strategic focal point of digitalization of metrology [21,59,60,61,62,63,64,65,66,67,68,69,70,71,72,73,74,75,76,77,78,79,80]. They are classified according to the four domains concerning this category. They are: (i) metrological analytical methods for data handling, storage, security and reliability (D2.1); (ii) metrological traceability in IoT (D2.2); (iii) use of cyber–physical systems, cloud computing, digital twins, artificial intelligence and machine learning in digital metrology (D2.3); and (iv) digital sensor network (D2.4).

The documents were analyzed, and the results are synthesized below, following the domain structure.

#### 4.2.1. Metrological Analytical Methods for Data Handling, Storage, Security and Reliability (D2.1)

Trust in the digital innovation relies on trust in the data, starting in the data gathering, data storage and the algorithms used to transform the input into something useful. Nowadays, the large number of sensors and the use of unstructured data (such as imaging processing for medical or additive manufacturing purposes) produce a significant amount of data, forcing big data analysis as an imperative to meet the requirements for quality assurance in novel digital procedures [1,2].

Big data are broken into five dimensions: (i) volume (massive amount of data); (ii) velocity (speed of data exchange); (iii) veracity (the degree of trust); (iv) variety (range of data types and sources); and (v) value (business value collected). Digital metrology mainly depends on the veracity dimension.

The connection between the measurement and the evaluation of the measurement data is becoming closer and closer, leading to the growing importance of mathematical and statistical procedures. The metrological analytical methods to handle this rely on data analysis, statistical learning, simulation and uncertainty analysis through fitting and optimization methods, inference and dimensionality reduction, where the structures existing in the data are exploited in a targeted manner to reduce the amount of data while maintaining the same content in terms of information or surrogate methods [1,2].

Emmer et al. [52] state that an early, faster and convenient procurement of measurement data (including geometry and related product manufacturing information) is crucial for production planning, and an intelligent measurement data management (MDM) enables enhanced automation of operations, consistent quality control, increased efficiency of single process steps and improved early risk identification. Despite the continuous trend toward digital metrology, seamless data continuity and exchange, and the massive amount of real-time data, some information is reported incompletely and inconsistently, particularly in measurement planning, disaccording with the five dimensions of big data. Emmer et al. [54] introduced a novel approach for a comprehensive MDM that fulfills the technological requirements of Industry 4.0 for complex process chains: (i) reduction in conversion-related data losses; and (ii) reduction in the costs of specialized employee training.

McGregor et al. [69] ran into the volume of big data’s dimension and proposed an automated method for batch processing X-ray computed tomography (CT) metrology data in additive manufacturing (AM) processes. Batch processing of CT data can be challenging and time consuming, mainly due to part complex geometries created by AM. That part could result in over 1000 measurements. At the same time, CT is the only non-destructive inspection technique capable of measuring internal features inaccessible to optical or tactile measurement.

The most straightforward task of on-machine surface metrology is to replace the conventional post-manufacturing inspection of the work piece surface carried out on an off-machine and stand-alone surface measuring instrument, so as to address data quality control in surface metrology. In this context, Gao et al. [63] described the state-of-the-art on-machine and in-process measurement systems and sensor technologies, overviewed the error separation algorithms for removing machine tool errors and discussed calibration and traceability. Additionally, they demonstrated some advanced techniques for sampling strategies, measurement systems–machine tools interface, data flow, analysis and feedback for compensation manufacturing.

D’Emilia and Gaspari [56] developed a methodology aiming at introducing validation actions in all steps of the measurement process in a “big data” scenario for Industry 4.0: (i) measurement; (ii) data retrieval; (iii) data storage and organization; (iv) data processing; and (v) data presentation. In this way, the variability of results and, consequently, their uncertainty could be reduced. The authors applied the proposed methodology in a real scenario using a neural network to classify some features in an industrial process and verify that this methodology allowed realizing a better feature extraction for the classification algorithm.

#### 4.2.2. Metrological Traceability in IoT (D2.2)

As defined in Section 4.1.2, metrological traceability is “a property of a measurement result whereby the result can be related to a reference through a documented unbroken chain of calibrations, each contributing to the measurement uncertainty” [96] (p. 29). The internet of things (IoT) is an interrelated intelligent device system that uses embedded systems to gather or transmit data from their environments. This technology has excellent engagement and has been widely used in many fields, including in industries, wherein it is called the industrial internet of things (IIoT), which uses these intelligent sensors and actuators to enhance the manufacturing and industrial processes. IoT helps construct a platform for sharing and interconnecting all kinds of manufacturing metrology resources and is usually formed by: (i) hardware (sensor, actuators or transmitter devices); (ii) middleware (data storage or data processing); and (iii) data visualization (to present the data) [64].

Gao et al. [63] discussed the metrological traceability forms when presenting in-process measurement systems, namely: (i) machine metrology traceability; (ii) machining metrology traceability; and (iii) machined surface metrology traceability. The first concerns the machine that does the job, while the second talks about the element that shapes the surface and determines the surface state in terms of the form and texture. The last is used for monitoring or measuring the surface directly and is the most relevant in general.

IoT devices are low-cost and low-computing-power devices, so they cannot process all the generated data [64]. Based on that fact, the generated data are stored and processed in the cloud using cloud technology. In this regard, Majstorovic et al. [64] proposed a module to address the connection, communication, computing and control of all metrology process levels from IoT to the cyber–physical manufacturing metrology model (CPM^3^).

#### 4.2.3. Use of Cyber–Physical Systems, Cloud Computing, Digital Twins, Artificial Intelligence and Machine Learning in Digital Metrology (D2.3)

Comparable to trust in data, trust in algorithms or methods is an absolute prerequisite for sustainable and reliable applications. Therefore, the main focus is always on ensuring reliability and trust in the results of the algorithms by consistently incorporating measurement uncertainties and data quality [1,2].

Cyber–physical systems (CPS) are a new generation of systems with integrated computational and physical capabilities that can interact with humans through many new modalities that far exceed today’s levels of autonomy, functionality, usability, reliability and cyber security. Interacting with and expanding the physical world’s capabilities through computation, communication and control is crucial for new technology developments [102].

Cloud computing is a term to describe a platform that dynamically provisions, configures and reconfigures servers as needed. The cloud infrastructure can be a cost-efficient model for delivering information services, reducing resource management complexity, promoting innovation and increasing responsiveness through real-time workload balancing.

Artificial intelligence (AI) is an integration of computer science and physiology. This term relates to any technique enabling computers to mimic human intelligence using simple or complex algorithms, such as decision trees, if–then rules or neural networks to recognize patterns, make choices, adapt to change and learn from experience [103]. In this regard, machine learning (ML) is a subset of AI that includes statistical techniques and mathematical models to enable machines to improve tasks with experience and make predictions or decisions without being explicitly programed to perform the task [104].

Cyber–physical systems (CPS) can be integrated with the internet of things (IoT) and cloud computing to generate cyber–physical manufacturing systems (CPMS). They represent a high-tech methodology for developing a new generation of factories with ever-increasing intelligence, flexibility and self-adaptability. However, generating detailed process models from quality measurements in manufacturing requires the development of a dedicated framework [102]. Majstorovic et al. [53] proposed the cyber–physical manufacturing metrology model (CPM^3^) based on integrating digital product metrology information through metrology features recognition and generation of a global/local inspection plan to address this case. In other words, CPM^3^ aims to enable the integration of massive amounts of rapidly obtained product metrology data with data-mining capacities that enable quick, adaptative process control and operations planning. In 2018, they faced several issues referring to the big data analysis, such as extracting useful information from data sets and finding the relevant structure from unstructured data sets, as described in Majstorovic et al. [55].

One year later, Majstorovic et al. [64] proposed an IoT model application for CPM^3^ to address the connection, communication, computing and control of all metrology process levels. Similarly, Sabbagh et al. [65] worked to enable a cloud-based model for big data analytics within the CPM^3^ utilizing optical metrology data linked and stored on a cloud, dealing with the problem of curating metrology data required for CPM^3^. They developed a method for model-based compression and distance-based organization of metrology data to address this. Later on, Sabbagh et al. [67] proposed a novel data curation concept that enables data mining and analytics within the CPM^3^ based on organizing the metrology data into tree-based database structures using distance-based unsupervised clustering of the raw metrology data, which enables logarithmic acceleration of searches within the data and thus provides expressive improvements for data mining.

Anwer et al. [57] addressed the classification of partitioning operations to provide a science-based solution for developing ISO Geometrical Product Specifications and Verification (GPS) partitioning standards. In the ISO GPS standard, partitioning aims at decomposing a part into independent features or surface portions for further processing and analysis.

Still, in CPS applications, robotics can boost productivity in the 3D measurement of components. However, such systems use expensive optical trackers or photogrammetric cameras to estimate the transformation matrix between multiple views [58]. To address this, Rao et al. [58] presented the development of a 3D scanner using simple and automatic registration techniques that do not require costly equipment for pose estimation.

Berry and Barari [59] employed CPS to extract information from the “work-piece memory”, a concept also introduced in this paper, which is the use of the workpiece as a source of information in contrast to on-machine inspection systems, aiming to control the production process.

Concerning the full integration of machines and production systems with machine-learning methods to enable intelligent multistage manufacturing, Papananias et al. [60] discussed the multistage manufacturing processes (MMPs). Under this framework, the machines and production systems can share data and information, detect manufacturing errors and poor quality in machined parts and take corrective actions to minimize part variation and propagation. In addition, the authors present an intelligent metrology informatics system to extract useful information, such as temperature, material conditions, force and vibration from MMP data, and predict part quality characteristics using a multi-layer perceptron (MPL) neural network supporting time-effective extraction. Papananias et al. [62] also contemplated MMP and presented a Bayesian linear regression model to estimate the results of post-process inspection from in-process monitoring data.

Nasir and Sassani [21] argued that the emerging topics on big data analytics, information fusion, data mining and ML/DL models have dramatically changed the state of the art in intelligent machining and tool monitoring toward data-driven approaches. In this regard, they reviewed the main applications, opportunities and challenges associated with data-driven approaches focusing on DL models. They identified and discussed opportunities in this field, such as automated feature engineering, handling big and high-dimensional data, avoiding sensor redundancy, optimal sensor fusion and hybrid intelligent models. They also pointed out critical data-driven challenges in smart manufacturing, including those associated with the data size, data nature, model selection and process uncertainty.

Machine learning needs high-quality data to learn and improve a process, but industrial data face some challenges, such as class imbalance, data drift and the lack of trained feature extractors [103,104]. To overcome these challenges, Tnani et al. [72] presented an efficient two-stage feature-learning approach that bridges the gap between unsupervised learning and few-shot learning, which makes it suitable for the industrial scenario where a large quantity of sensory data is available with a limited number of labels. This new method shows higher generalization capabilities than the traditional prototypical network.

A digital twin (DT) is the virtual replica of a real-world product continuously updated with data from its physical counterpart and environment. It bridges the virtual cyberspace with physical entities and, as such, is considered to be the pillar of Industry 4.0 and the innovation backbone of the future [105].

Using DTs, Gohari et al. [61] presented a virtual replica to work in parallel with an integrated inspection system (IIS) to inspect freeform and complex surfaces based on a metric of their geometric complexity to reduce the inspection time and uncertainties in the evaluation of the substitute geometry and estimation of the deviation zones.

Moyne et al. [66] proposed a baseline framework for DT technology that leverages the knowledge gained from developing existing DT solutions and incorporates the requirements placed on DT technology by smart manufacturing trends and the ultimate DT vision. The framework includes a definition of an object-oriented architecture for DTs that incorporates the bottom-up knowledge gained from practical development and implementation of today’s DT classes while addressing requirements such as re-usability, extensibility, interoperability, interchangeability and autonomy.

Due to a rapid increase in the use of collaborative robots in manufacturing industries within the context of Industry 4.0 and smart factories, Gallala et al. [71] developed a digital twin (DT) approach for human–robot interactions (HRIs) in hybrid teams, since the existing HRIs are time consuming, require engineering expertise, waste a lot of time on programing, and the interaction is not trivial for non-expert operators. The application of the proposed DT indicated that it has further benefits, such as real-time simulation in natural environments, no requirement for pre-trained operators and flexible system integration to incorporate new devices.

Choi et al. [73] proposed a DT architecture based on an interoperable data model, aiming at continuous collaboration between field engineers for data gathering, designers for modeling 3D models and layout engineers for layout changing by generating 3D digital twin models automatically. The authors provided examples applied to the Korean automotive parts manufacturers.

Using cloud computing and virtual reality to mitigate the manufacturing accuracy influence of external and internal factors, Stepanek et al. [70] discussed the critical aspects in implementing Industry 4.0 with a focus on metrology to ensure long-term production accuracy of CNC machine tools.

#### 4.2.4. Digital Sensor Network (D2.4)

The importance of reliable and accurate measurement and positioning in large volumes in the manufacturing industry has increased significantly [68]. These measured data majorly come from devices that detect and respond to inputs from the physical environment, called sensors. With IoT, sensor networks—a group of sensors where each sensor monitors data in a different location and sends those data to a central location for storage, viewing and analysis—are increasingly used in the industry.

In this regard, Jia et al. [68] proposed a rapid and flexible calibration method based on the highly precise three-dimensional coordinate control network, including estimation and optimization algorithms, using a large-scale positioning system due to its capability to increase the number of transmitters and sensors to expand the workspace and ensure positioning accuracy, becoming a faster and easier solution due to its high-accuracy, real-time, easy expansion and multitasking characteristics.

### 4.3. Metrology of the Communication Systems for Digitalization

Table 6 presents a summary of the documents reviewed in this category [74,75,76,77,78,79,80,81,82,83,84,85]. They are analyzed according to the following domains: (i) data-based metrological infrastructure in complex industrial scenarios (D3.1); (ii) integration of IoT, cyber–physical systems and cloud computing for efficient communication (D3.2); (iii) national digital quality infrastructure (D3.3); and (iv) traceability, conformity assessment and standardization (D3.4).

#### 4.3.1. Data-based Metrological Infrastructure in Complex Industrial Scenarios (D3.1)

New digital technologies are designed to maximize efficiency, enable economies of scale and develop new services. So, it is necessary: (i) to develop an infrastructure to support the processes of conformity (a trusted metrological core platform); and (ii) to define requirements for machine-readable data exchange in digital communication [74].

As mentioned in Section 4.1, a pan-European consortium led by PTB has initiated the development of a coordinated European DQI for new products and services called the “European Metrology Cloud” (EMC) [11]. The term cloud here is interpreted as a synonym for the trusted metrological core platform, which is distributed and designed to support the processes of conformity assessment and market surveillance/verification and the development of reference architectures and new technology data-driven services for this infrastructure [75].

The major impacts of this DQI are: (i) the establishment of a metrological trust anchor; (ii) the easy integration of contributors, data and infrastructure; (iii) digitally rendered workflows; (iv) digitally streamlined metrological processes; (v) harmonization of processes by technology; (vi) the possibility to repair/verify a large number of measuring instruments remotely; (vii) digital support services that reduce the downtime of the measuring instrument; (viii) globally available measuring instrument data (manuals, certificates, among others); (ix) measuring instrument data open to integrating concepts from other regulated areas; and (x) measuring instrument data open to integrating other trust-based workflows [75].

From the perspective of reducing the ambiguity and incorrect interpretation caused by missing metadata and diversity of units, among others, Ačko et al. [78] described a formal framework for the transmission of metrology data based on the SI within the scope of the European Project EMPIR 17IND02 SmartCom, which was agreed between the European Commission and the European Association of National Metrology Institutes (Euramet). According to the authors, SmartCom is one of the first projects in metrology to define the universal minimum requirements for machine-readable data exchange in digital communication. Furthermore, it provides a basis for representing data in future digital applications in metrology, such as data for metrological services and data exchanged between virtual measuring instruments. DCCs are a virtual representation of the properties of measuring artifacts and instruments [78].

Paciello et al. [79] specified a harmonized, universal, coherent machine-readable uniform metadata model for the digital transmission of complex quantity information in machine-to-machine communication, following the specifications of the International System of Units. The conventions for evaluating and expressing measurement uncertainty are based on the internationally recognized GUM [98]. The metadata model developed within the SmartCom project provides atomic and extended representations of a complex quantity (for Cartesian or polar form, following the guidelines of GUM S2) [79].

#### 4.3.2. Integration of IoT, Cyber–Physical Systems and Cloud Computing for Efficient Communication in Complex Industrial Scenarios (D3.2)

A shift from the current internet of things (IoT) to include more industrial equipment and metrology systems is forming the industrial internet of things (IIoT). Metrology measurements of the IIoT sensors working in critical infrastructures can be essential and even affect the safety of human lives. However, hackers have targeted industrial sites, which are subject to cyberattacks, bringing many concerns related to confidentiality, integrity, availability, privacy and non-repudiation. Another concern is an existing gap in metrology device integration (interoperability) [74]. Moreover, it is necessary to define some requirements for machine-readable data exchange in digital communication. So, the metrology measurements of the IIoT sensors bring some challenges related to (i) the data security produced, (ii) the interoperability among devices and (iii) the minimum requirement in digital communication [78].

From this perspective, it is important to develop IIoT technologies to secure communication and resilient wireless networks to protect industrial data and safely store industrial intellectual property in cloud systems [74,78,85]. Therefore, there is a demand for custom security solutions, rather than generic ones, ensuring the security of the IIoT value chain [74]. For example, considering the IIoT trust for DCCs, a digital signature can be created using public cryptography. The signee must have a mathematically cryptographic key pair of public and private keys [78].

By applying the theory of constraints (TOC), Chen et al. [85] developed a set of IoT controllers with integrated wireless transmission and functions to enable users to control programable logic controllers (PLCs) more easily. This research used the TOC to identify the bottlenecks of current PLCs and then addressed these limitations to develop improvement measures to remove them. These measures include: (i) modular human–machine interface (HMI) operations; (ii) wireless transmission; and (iii) real-time messaging.

In an attempt to customize a solution for devices for smart meter infrastructures, Peters et al. [77] proposed a framework using homomorphic encryption (HE) combined with blockchain technology to achieve confidentiality for those distributed measuring instruments. Misbehavior of these devices can lead to a considerable loss of money for all the stakeholders. Since software is one of the critical components of such devices, and developments in the overall information technology market, such as cloud computing and the internet of things, are progressing into the legal metrology market, we can expect measuring instruments to become more and more integrated into open networks.

As already explained, cloud computing, big data, artificial intelligence and the IIoT are designed to maximize efficiency, enable economies of scale and develop new services, bringing benefits to the industry, such as agility, productivity, speed of deployment and autonomy. By way of illustration, in the legal metrology context, since digital system architectures, digital services and digital infrastructures must be legally compatible with the society’s demands, stakeholders (such as industries, notified bodies and market surveillance/verification authorities) would be rewarded with better communication legal processes [75].

Considering the necessity to minimize the problem in metrology device integration (interoperability), Sousa et al. [84] proposed a generic information model in which measuring devices can have their data collected through a generic open platform communications unified architecture (OPC UA) interface to participate in the IEC 62264 Quality Operations Management activities, enabling integration with upper systems, such as enterprise resource planning (ERP), and the creation of quality-oriented key performance indicators (KPIs). An experimental scenario in the steel manufacturing industry was conducted, where authors demonstrated how a generic interface could support custom software applications by using metrology data support, resulting in a reduction in product and process defects.

The data model proposed by Paciello et al. [79] is expressed in extensible markup language (XML) and is based on the minimum requirements for the machine-readable exchange of metrological data that are described in the “Digital System of Units” (D-SI) metadata model, developed within the European EMPIR Project 17IND02—SmartCom (see Section 4.3.1).

Accordingly, Ačko et al. [78] presented the digital system of units (D-SI) metadata model, which can help developers of data formats to implement their data in an unambiguous, easy-to-use, safe and uniform way that is based on the SI and other internationally accepted guides, providing a data basis for representing data in future digital applications in metrology. The authors affirm that big data analysis is also facilitated if the data are based on common terminology in metrology. DCCs are a very important application of the metrological data exchange, using the principles from the D-SI, XML as a machine-readable format, and fundamental requirements from the ISO/IEC 17025 standard [100].

As posed by Nummiluikki et al. [81], to build a shared DCC platform solution, some aspects must be considered: (i) to agree with a standard DCC within the calibration system partners; (ii) to assure that the industry improves data integrity and process efficiency of their calibration management; and (iii) to diminish the barriers to putting DCCs into use, at all levels of digitalization, and resources of an individual calibrator or instrument owner.

According to Ref. [81], DCCs alone will not enable all possibilities of digitalization; a fully automated calibration that enables automated communication of DCCs needs to be interoperable. In this regard, it is necessary to define the basic principles for exchanging machine-readable data to address interoperability. Some essential requirements should be stated: (i) every quantity expressed must comprise at least one value and a corresponding unit (atomic-quantity-type representation); and (ii) a vast-quantity-type representation is introduced to add information about the uncertainty of measurement to the atomic quantity type. This representation introduces the expanded uncertainty associated with the measured value, the coverage factor, the coverage probability, and optionally, the distribution.

In the future, DCCs will record all aspects of the calibrated items and make them available to a comprehensive quality management system. With these complete data sets, the performance of the systems and processes can then be captured effectively and efficiently, allowing data analytics methods to provide information on optimized system performance. This activity leads to reduced downtime, less waste, significant improvement in quality, and ultimately, greater economic success [81].

#### 4.3.3. National Digital Quality Infrastructure (D3.3)

A digital system structure is important for developing metrology for digital transformation at NMIs. In this regard, the implementation of an inter-NMI blockchain network is addressed to deliver connecting NMIs (peers) worldwide [94], since blockchain-based public key infrastructure (PKI) could gain significant benefits from using digital signature, providing integrity, authenticity and non-repudiation of legal metrology information [76,80,83].

A plan, which includes the digitalization of metrology standards, measuring instruments, metrological information and metrological activities for the digital transformation, is proposed for the National Institute of Metrology (NIM) in China [82]. NIM will give priority to the development of mapping regarding VIM [96] and GUM [98], a general digital calibration certificate metadata model and supporting software. In this regard, Xiong et al. [82] proposed a framework that will be improved in an iterative way in order to achieve a comprehensive digitalization of metrological information, including a traceable chain of metadata models for metrological information, conversion and verification software libraries and a supporting digital infrastructure.

Legal metrology plays a crucial role by providing confidence in the measurement of physical quantities, and it must take advantage of the digitalization of metrological activities. The EMC initiative is a good example. Moni et al. [83] discussed the main aspects and features that an inter-NMI blockchain network must deliver, connecting peers from the PTB (Germany) with NMIs from other countries. This initiative started with a joint action of two NMIs: the PTB and the National Institute of Metrology, Quality, and Technology (Inmetro), respectively—the German and Brazilian NMIs [16]. The proposed architecture consists of a blockchain-based public key infrastructure (PKI) that meets the main requirements imposed in legal metrology and opens up the opportunity to integrate PTB with NMIs from other countries [16,83].

As concept proof, Moni et al. [83] demonstrated the use of DCCs in smart metering systems could benefit from using digital signatures of a blockchain-based PKI as a mechanism to provide the integrity, authenticity and non-repudiation of legally relevant information. Blockchain-based PKI is a promising alternative to improve the reliability of smart meters once conventional PKIs become too expensive in scenarios that involve a significant number of meters [83]. Melo et al. [76] implemented a vehicle speed measuring system using the Hyperledger Fabric blockchain platform and discussed how blockchains can be used to support the distributed measuring systems. Due to their security properties, blockchains can improve metrological assurance by imposing restrictions against potential attacks while reducing technical efforts related to regulation and control activities.

Considering a national DQI as a scope, Thiel and Wetzlich [75] analyze how recent regulations within the digital single market strategy of the European Commission—the Data Protection Police Directive (GDPR) (2016/679/EU) and the Regulation on a framework for the free flow of non-personal data in the European Union (Regulation (EU) 2018/1807)—may be integrated into the “European Metrology Cloud” initiative to, e.g., guarantee that its underlying blockchain approach complies with these norms and exploits their benefits.

Metrological regulation and control require more efforts from notified bodies, becoming slower and more expensive, and blockchain has been used as a resource to overcome such challenges by the NMIs [76]. However, European Commission is monitoring whether the legal framework is sufficient to allow further development of blockchain technology and its applications in the EU. Regarding the GDPR, for example, the recommendation is to avoid storing personal data on a blockchain; personal data should be carefully considered when connecting private blockchains with public ones [71]. Instead, entities should make full use of data obfuscation, encryption and aggregation techniques to make data anonymous. If blockchain cannot be avoided, the recommendation is fulfilled by the metrology cloud approach with permissioned blockchain [75].

So, the metrology cloud benefits from these new regulations, making it future proof, which is in line with the digital market strategy of the European Commission, the Data Protection Police Directive and the Regulation on a framework for the free flow of non-personal data in the European Union.

#### 4.3.4. Traceability, Conformity Assessment and Standardization (D3.4)

Industry 4.0 has been using multiple sensors that measure all aspects of the production process, resulting in a complete set of measured data used to understand, in much greater detail, the performance of the system, leading to reduced downtimes, fewer rejected parts, improvements in quality, better-organized maintenance, better conservation of energy and resources, and increased business success. However, the quality of the measurement information through the metrological concepts of traceability and uncertainty must be pursued. In this sense, industrial metrology is increasing its awareness of its central role in Industry 4.0 for the reliable continuous data collection on the quality characteristics of an item, either a product or a process in the scope of quality assurance, and receiving a demand for enhancing integration, interoperability and availability of measurement information for other operations [84].

Big data analysis is also facilitated if data are based on common terminology in metrology. In the future, DCCs will record all aspects of the calibrated items and make them available to a comprehensive quality management system, leading to reduced downtime, less waste, significant quality improvement, and ultimately, economic success [78].

Within the legal metrology field, for example, the digital transformation shall remove barriers to innovation within the legal processes and reduce costs and time to market for new digital products. In this sense, the “European Metrology Cloud” (EMC) [11] aims to support the processes of conformity assessment, market surveillance/verification and the development of reference architectures, new technology and data-driven services for this infrastructure [75].

### 4.4. Metrology for Simulations and Virtual Measuring Instruments

Table 7 summarizes the contributions of 11 documents related to this focal point [19,23,86,87,88,89,90,91,92,93,94]. A qualitative analysis of the abstracts and full texts of the referred documents enabled the identification of the three main domains concerning metrology for simulations and virtual measuring instruments. They are: virtual metrology (D4.1); deep learning and digital transformation of metrology (D4.2); and augmented reality and digital transformation of metrology (D4.3).

#### 4.4.1. Virtual Metrology

Virtual metrology (VM) was originally defined by Weber [106] (p. 52) as “estimating values for metrology (i.e., substrate feature measurements) by using equipment/process parameters and other available production data which may include values from previous metrology steps as well as production context information”. Recently, it has experienced a renewed interest in the context of Industry 4.0 and the digital transformation of metrology, as highlighted by the documents reviewed in the present SLR.

Tieng and his research group have developed an automatic virtual metrology (AVM) system [107] for predicting the machining precision of machine tools, consisting of four parts: data preprocessing, data quality evaluation, feature dimension reduction and machining precision prediction. It can automatically evaluate data quality, provide the predicted precision with a reliance level and allow the prediction model to be tuned or re-trained. Later on, in 2017, this research group coupled the AVM to another scheme, called the target value adjustment (TVA), designed to enhance the AVM’s adaptive customization capability in order to facilitate the migration from mass production toward mass customization (MC), encompassing large scale, low cost, short lead time and high quality [86]. One year later, the same research group coupled AVM with yet another scheme, called deformation fusion (DF) [87], for dealing with component deformation problems in the manufacturing of complex aerospace components, which suffer from long metrology delays and severe deformations. Interestingly, the DF scheme estimates the deformation information without adding massive machining cycle time.

Maggipinto and colleagues [88,89,90] have also worked with virtual metrology, specifically in the semiconductor manufacturing domain. They have studied in depth the problem of feature extraction in multidimensional data related to VM, aiming to improve the manufactured parts’ classification. Hou et al. [92] have developed a similar work, also in the semiconductor manufacturing industry, specifically aiming at the detection of defects in chips.

Hsieh et al. [93] also worked with AVM, proposing a production data trace-back (PDT) mechanism that provides a virtual label to each unit produced in a continuous production process for carbon fiber components. Their goal was a real-time total quality inspection in continuous production processes, and they also adopted the Advanced Manufacturing Cloud of Things (AMCoT) platform to fulfill the goal of zero defects in carbon fiber manufacturing.

Dreyfus et al. [19] have performed an SLR in VM, including an integrative conceptual framework, aiming at the application of product quality estimation in smart manufacturing. Finally, Chien et al. [94], also working in the semiconductor manufacturing domain, developed a decision-based VM framework that integrates clustering and regression models to enhance the prediction and ensure the decision quality in the production process.

Considering the documents related to the research literature on VM, it is clear that the main application of the technique is in quality assurance, aiming to achieve a faster and more reliable classification scheme for the manufactured parts online and in real time.

#### 4.4.2. Deep Learning and Digital Transformation in Metrology

Deep learning (DL) techniques have undergone enormous growth in the past few years in several application areas, including Industry 4.0 and the digital transformation of metrology, mainly because of their powerful representational power and classification capabilities. Unlike classic machine-learning (ML) approaches, DL algorithms are able to deal directly with raw input data without the need for a feature extraction phase, which is frequently the most difficult component of an ML approach. DL algorithms are based on multilayer neural networks (hence “deep”) that are able to automatically extract increasingly complex peculiarities of the data.

Maggipinto and colleagues [88,89,90], who have worked with VM in multidimensional classification problems, as explained in the previous section, have employed several DL algorithms to automate the feature extraction in the semiconductor manufacturing domain.

Another contribution came from Hou et al. [92] who developed a system for automatic visual inspection of micro-defects on semiconductor laser chip surfaces based on virtual metrology (VM) resources and deep learning (DL) tools to handle the large volume of data that need to be classified.

All the identified applications of DL in the digital metrology arena are related to feature extraction from large volumes of data, specifically with the end goal of quality assurance of the manufactured parts, coupled with virtual metrology schemes, as depicted in the previous section.

#### 4.4.3. Augmented Reality and Digital Transformation in Metrology

Augmented reality (AR) enjoyed its first real application in the industry in the early 1990s when scientists at the Boeing Corporation developed an experimental AR system to help workers put together wiring harnesses [108]. The basic idea is to superimpose computer-generated images on real-world images, normally with the aid of a digital imaging device or tailor-made goggle that the operator wears. It has been widely used recently in several fields, such as military, industrial and medical applications, commercial and entertainment use.

Ferraguti et al. [91] have explored the application of AR in the quality assurance scenario in order to support the assessment of polished surfaces’ quality. Initially, the polished surface undergoes surface measurement, based on contact or non-contact systems. The former includes tactile instruments (contact profilometer, atomic force microscopes), and the latter includes optical instruments, such as the ones covered in the previously mentioned SLR conducted by Catalucci et al. [22]. Then, the surface metrology data are directly projected on the polished component surface through an AR headset worn by the operators, so both the end user and the operator can directly see on the component if the quality satisfies the specifications or if some parts of the surface require further refinements.

Taking a broader approach, Ho et al. [23] presented a SLR on AR systems and their applications in the smart manufacturing context from 2010 to 2021. They showed that there is a tendency toward interest in developing and implementing AR-assisted quality applications, currently considering three main categories: AR-based apps as a virtual lean tool, AR-assisted metrology and AR-based solutions for in-line quality control. The paper by Ferraguti et al. [91] falls into this last category.

Similar to the domains of virtual metrology and deep learning, the main focus of the application of augmented reality in the context of digital metrology is on automating and accelerating quality assurance tasks, often coupled with other techniques.

### 4.5. Discussion

From the results of an in-depth literature analysis of 70 documents presented in this section, it was possible to summarize the most significant challenges and latest trends concerning the digital transformation of metrology, organized into four categories and 16 domains that integrate the analytical framework adopted in this review. So, the first two research questions defined in the introductory section are re-addressed here.

#### 4.5.1. Digital Transformation of Metrological Services

Focusing initially on the implementation of DCCs, it was argued in Ref. [48] that these certificates will soon replace paper calibration certificates (PCCs), and this transition will require a digital security and software infrastructure to fulfill the authentication and validation processes that deliver the whole traceability chain to primary standards. By reviewing the documents more directly related to the research literature on DCCs [17,33,41,48], it became apparent that some research works concentrate on the reliability validation of these certificates and their metrological traceability [32,40,46]. In contrast, other works have a broader scope to create a complete measurement infrastructure in which DCCs represent only one part, as discussed in Refs. [17,34].

The importance of blockchain-based applications in the context of digitalization in metrology has been strongly emphasized. From the findings presented in Refs. [32,36,41,42,51], it is argued that blockchain-based technologies have multiple properties that match the needs concerning the digital transformation of metrology. For example, these technologies can enable metrological traceability to the highest level of the chain, providing calibration history by preserving machine-readable records, among other benefits. Nonetheless, since trust in the measurement results requires a holistic approach, it is necessary to coordinate the blockchain applications and development in the transition toward digital metrology to accomplish long-term self-sustainable solutions.

Regarding the concerns on the digital representation of physical quantities and units of measurement, the demand for a new digital measurement infrastructure was discussed in Refs. [37,43,50] and illustrated briefly by the need for a worldwide agreement on standardized machine-readable formats for the SI, the representation of measurement results and the secure authentication, transfer and use of measurement data, including DCCs, as discussed in Section 4.1.1.

Digital representation of metrological traceability was discussed in Refs. [36,38,47], and their findings suggested that, while the GUM Supplement 1 (GUMS1) approach to uncertainty evaluation based on the Monte Carlo method is well established [98], the potential overlap between GUMS1 and DCCs should be more investigated. Furthermore, the representation of measurement uncertainty in digital records should not follow the current practices. Preferably, the reporting formats should include uncertainty components wherever possible because digital systems can use this information in end-user applications.

From the view of digitalization in legal metrology, the findings reported in Refs. [30,31,47] indicated the high potential of blockchain-based applications in this context, particularly those for measuring instruments under legal control. Some promising areas were highlighted in Refs. [28,45]—for example, the complete automation of smart contracts’ legally supervised update mechanism and the integration of measuring instruments in the distributed measuring systems (DMS). Parts of the works reviewed on this topic [28,35,39,45,49] were more concerned with the institutional role of NMIs in fostering the digital transformation in legal metrology in European countries. However, they also pointed out that digital transformation of metrological services, in general, should lead to significant cost savings in national quality infrastructures. Finally, one of the reviewed studies highlighted essential aspects concerning the implementation of a national DQI in India, based on a SWOT analysis, over the next few years [44].

Mindful of the fact that most of the focus of the scientific contributions reviewed in Section 4.1 is on blockchain-based methods, the architectural and protocol aspects of centralized and institutional-based metrological systems are re-addressed and discussed more deeply in Section 4.3 and Section 4.5.3.

#### 4.5.2. Metrology in the Analysis of Large Amounts of Data

The main challenge of metrology in analyzing large amounts of data consists of developing the metrological analytical methods for assessing ML and DL models and big data analytics. Despite the big opportunities concerning the use of ML and DL models, there are still challenges facing a data-driven industrial approach, especially concerning the size and quality of the acquired data. In Ref. [21], the challenges associated with the data-driven approach were categorized into four main groups, as follows: (i) data size challenges; (ii) choice of DL models; (iii) data nature challenges; and (iv) uncertainty associated with the DL models generated on a laboratory scale.

It is possible to verify that two domains are most investigated in this category. They are “Metrological analytical methods for data handling, storage, security and reliability” and “Use of cyber–physical systems, cloud computing, digital twins, artificial intelligence and machine learning”. The first is captained by the dimensions of big data, with scientific publications mainly focused on real-time massive amounts of data processing, data batch and data veracity [52,54,56,63,69]. At the same time, CPS technologies, especially when integrated with IoT and cloud computing to generate CPM^3^ [53,55,64,67], have stimulated a new generation of factories with ever-increasing intelligence, flexibility and self-adaptability.

Research on digital twins has been conducted intensively worldwide. They have been built and utilized in: (i) digital model building; (ii) real-time field data connection; (iii) monitoring and analysis; (iv) stakeholder decision making; and (v) field application procedure [73]. Regarding the interface with the digitalization of metrology, new developments as proposed by Ref. [73] are addressed to improve the continuous collaboration between field engineers for data gathering, designers for modeling 3D models and layout engineers for layout changing by generating the 3D digital twin models automatically.

#### 4.5.3. Metrology of the Communication Systems for Digitalization

Concerning the challenges associated with metrology of the communication systems for digitalization, this review pointed to the need to develop a communication infrastructure to support the conformity processes, called a trusted metrological core platform. Moreover, the necessity for creating some requirements for machine-readable data exchange in digital communication was reinforced in Refs. [75,78].

There is a demand for custom security solutions to ensure the security of the IIoT value chain [74]. The use of homomorphic encryption combined with blockchain-based technologies [75,77,80,82,83] could be a solution to achieve confidentiality for those distributed measuring instruments. IIoT controllers with integrated wireless transmission and PLC functions have been investigated to enable users to control PLCs more easily, including modular human–machine interface (HMI) operations, wireless transmission and real-time messaging [85].

The necessity for the interoperability protocols is defining the principles for exchanging machine-readable data to address interoperability. An actual example of this is DCC, an application of the metrological data exchange, which uses the principles from the D-SI, XML as a machine-readable format and fundamental requirements from the ISO/IEC 17025 standard [78]. Another case is the use of open platform communications unified architecture (OPC UA) to solve the problem of metrology device integration [84].

The quality of the digital measurement within the concepts of traceability and uncertainty must be pursued. DCC, for example, could record all aspects of the calibrated devices and make them available to a quality management system. Moreover, big data analysis should be facilitated if data are based on common terminology in metrology [74]. In this regard, the “European Metrology Cloud” (EMC) aims to support the processes of conformity assessment and market surveillance/verification [75].

A digital system structure is important for developing the digital metrology for smart manufacturing at the level of NMIs [82]. Moreover, an inter-NMI blockchain network connecting NMIs (peers) around the world must be delivered [79]. PTB and the National Institute of Metrology, Standardization and Industrial Quality (acronym in Portuguese, Inmetro) in Brazil undertook an important initiative in this direction, which seems to be bringing good results [83].

#### 4.5.4. Metrology for Simulations and Virtual Measuring Instruments

Considering the documents related to the research literature on VM, it is clear that much effort has been devoted to the development of the VM area, particularly the automatic virtual metrology (AVM) schemes [19,86,87,88,89,90,92,93,94]. The main application of the technique is in quality assurance, aiming to achieve a faster and more reliable classification scheme for the manufactured parts online and in real time. It has been originally developed for the semiconductor manufacturing arena, but it could easily be adapted to other industries that are already embracing the Industry 4.0 paradigm.

Deep learning (DL), the artificial intelligence technique that has been enjoying the largest growth recently, both in terms of new algorithms and new applications, is also being increasingly employed in Industry 4.0 and for the digital transformation of metrology. Mostly, the identified applications of DL in the digital metrology arena are related to feature extraction from large volumes of data, specifically with the end goal of quality assurance of the manufactured parts, coupled with the virtual metrology schemes mentioned in the previous paragraph [86,87,88,89,90,92].

The main focus of the application of augmented reality in the context of digitalization of metrology is on automating and accelerating the quality assurance tasks, often coupled with other techniques [23,91]. The basic idea is that the operator wears a headset, so that they can directly see if the quality of the component satisfies the specifications or if some parts of the surface require further refinements on-line and in real time.

Finally, based on the most significant challenges, latest trends and initiatives concerning the digital transformation of metrology discussed in this work, it is important to highlight here the four main contributions or differentials of the present work as compared to the results of previous related works [4,17,18,19,20,21,22,23], namely: (i) a new systemic and strategic look at the current state of research focusing on the interplay between smart manufacturing and digitalization of metrology; (ii) linked to the first, a broader scope compared with previous reviews encompassing all four focal points of a strategic approach for the digitalization of metrology with implications for different sectors of the economy and public policies (as shown in Table 1); (iii) an analytical framework comprising four strategic focal points (categories) and 16 specific domains that emerged from the qualitative in-depth analysis focusing on documents of each category (see notes in Table 4, Table 5, Table 6 and Table 7); and (iv) updating of ongoing initiatives [6,7,8,9,10,11,12,13,14,15,16] addressing the digitalization of metrology at the national, regional or global levels.

## 5. Conclusions and Research Agenda

In this paper, an attempt was made to conduct a SLR on the interplay between smart manufacturing and digitalization of metrology, addressing four strategic focal points with implications for different sectors of the economy and public policies. In this regard, 70 published scientific articles from 2016 to 2022 were retrieved from the Web of Science and Scopus databases, selected and reviewed.

The objectives of this study were achieved, and the findings summarized and discussed in Section 4 make significant contributions to the overview of the state of the art of this emerging research field around four strategic focal points, namely: (i) digital transformation of metrological services; (ii) metrology in the analysis of large amounts of data; (iii) metrology of the communication systems for digitalization; and (iv) metrology for simulations and virtual measuring instruments.

The results shed light on how policy makers, researchers and practitioners can better face the various challenges associated with the digital transformation of metrology from a smart manufacturing perspective. The main conclusions associated with the first two research questions defined in the introductory section were formulated in Section 4.5, according to the strategic framework adopted in this review.

Regarding the third question, from the perspective of building a research agenda in this research field, more than 90 % of the reviewed documents suggested future directions to expand the knowledge base on challenges and trends regarding the digital transformation of metrology to fulfill smart manufacturing demands. Accordingly, further research suggestions can be summarized by category, as follows:

1. Digitalization of metrological services:
  - Definition of specific vocabularies for finding information through algorithms, such as vocabularies and standardized term lists with persistent identifiers (PID), which can support the creation of metadata that will be machine readable and accessible worldwide in a language-neutral manner;
  - Structuring a new digital measurement infrastructure addressing the need for a worldwide agreement on standardized machine-readable formats for the SI, the representation of measurement results and the secure authentication, transfer and use of measurement data, including DCCs; and
  - Development of methods and models based on blockchain-based technologies and distributed ledger technologies to digitally represent the processes between the partners in legal metrology and for the future mutual recognition of DCCs.
2. Metrology in the analysis of large amounts of data:
  - Development of CPM3 modules that would enable the generation of virtual models of the parts using measurement results and their connection to the downstream and upstream processes in a cyber–physical manufacturing system;
  - Fine tune the machine-learning or statistical models or use other algorithms to improve the algorithm trustiness, explicability and performance. Additionally, industrial test cases should be developed to evaluate algorithm performance in practice;
  - Expansion of the use of IoT in sensor network to increase data gathering to improve process accuracy; and
  - Investigation of batch process and edge computing for faster, less resource-demanding and less expensive massive data processing with big data from IoT devices.
3. Metrology of the communication systems for digitalization:
  - Investigation of sensor–software communication and the feeding of metadata into a data portal that will link different data sources focusing on the development of new processes and business models in smart manufacturing;
  - Design of a customized security architecture for a conceptual industrial internet of things (IIoT) setup, aiming to reduce vulnerabilities in communication systems; and
  - Creation of a research network to connect NMIs around the world to answer research questions associated with the main challenges identified in this SLR.
4. Metrology for simulations and virtual measuring instruments:
  - Improvement of the deep learning (DL) methods that are currently employed in the virtual metrology (VM) arena, both in terms of larger data sets and better training of the models;
  - As further development of VM, integration of other intelligent methods into the Advanced Manufacturing Cloud of Things (AMCoT), such as intelligent predictive maintenance (IPM) and intelligent yield management (IYM); and
  - In the context of augmented-reality-enabled quality assurance, the interaction between the operator and the robot should be enhanced, allowing a deeper interaction with the production process.

The findings presented in this review can help policy makers, researchers and practitioners by providing directions for the evolution of all the strategic focal points covered in this SLR. In addition, the suggestions for a research agenda herein can motivate new research projects and teaching activities related to the digitalization of metrology from the perspective of their potential use in different smart manufacturing contexts.

Finally, as discussed in this paper, policy makers can better explore the state of the art of the interplay between smart manufacturing and digitalization of metrology to define public policies aiming at improving their national DQIs by promoting the use of digital solutions based on cloud computing, digital twins, artificial intelligence, blockchain, augmented reality and machine learning for interconnected and virtualized measuring systems.

## Figures and Tables

**Table 1:** Reviews and bibliometric papers on the interplay between smart manufacturing and digital metrology.

Review or Bibliometric Paper	Publication Year	Focal Point(s)
Sousa and Almeida [4]	2021	FP2, FP4
Gadelrab and Abouhogail [17]	2021	FP1
Varhney et al. [18]	2021	FP1
Dreyfus et al. [19]	2021	FP4
Yang et al. [20]	2021	FP2
Nasir and Sassani [21]	2021	FP2
Catalucci et al. [22]	2022	FP4
Ho et al. [23]	2022	FP4
**Present paper**	**2022**	**FP1, FP2, FP3, FP4**

Note: FP1—Digital transformation of metrological services; FP2—Metrology in the analysis of large amounts of data; FP3—Metrology of the communication systems for digitalization; FP4—Metrology for simulations and virtual measuring instruments.

**Table 2:** Bibliographic data collection.

Description	Results
Documents	160
Sources (journals, books, among others)	97
Annual Growth Rate	34%
Keyword plus (ID)	316
Author’s keywords (DE)	625
Period	2016–2022
Average citations per document	6.2
Authors	525
Authors of single-authored documents	7
Authors of multi-authored documents	518
Single-authored documents	7
Co-authors per document	4.2
International co-authorships	27.4%
Collaboration index	3.4

**Table 3:** Clustering of the selected documents by category.

Category	Reference	Number of Articles
Digital transformation of metrological services	[17,29,30,31,32,33,34,35,36,37,38,39,40,41,42,43,44,45,46,47,48,49,50,51]	24
Metrology in the analysis of large amounts of data	[21,52,53,54,55,56,57,58,59,60,61,62,63,64,65,66,67,68,69,70,71,72,73]	23
Metrology of the communication systems for digitalization	[74,75,76,77,78,79,80,81,82,83,84,85]	12
Metrology for simulations and virtual measuring instruments	[19,23,86,87,88,89,90,91,92,93,94]	11
**Total**		**70**

**Table 4:** Documents reviewed in the “Digital transformation of metrological services” category.

Document	Year	Objective	Domain(s)
Thiel [29]	2018	To describe the development and implementation of the European Metrology Cloud [11], designed to support the processes of conformity assessment and market surveillance in Europe by technology- and data-driven legal metrology services.	D1.5
Peters et al. [30]	2018	To discuss the main applications of blockchain-based technologies for measuring instruments under legal control and identify promising areas, e.g., the complete automation of the legally supervised update mechanism by smart contracts.	D1.5
Melo et al. [31]	2018	To propose a blockchain-based model on the assumption that blockchain-based technologies can improve metrological assurance of measurement instruments by imposing restrictions against potential attacks while reducing technical efforts related to regulation and control activities.	D1.5
Takatsuji et al. [32]	2019	To propose a blockchain technology to allow the owner of a calibration certificate to visualize the chain of calibrations to the primary standards, i.e., the source of traceability. Prevention measures of falsification of the certificates are included to assure authenticity and integrity of the certificates and access control of them.	D1.2 and D1.1
Mustapää et al. [33]	2020	To propose a conceptual solution based on DCCs, D-SI and cryptographic digital identifiers to validate data quality, authenticity and reliability. The data that enable validation and traceability can improve analytics, decision making and security in industrial applications.	D1.1
Brown et al. [34]	2020	To present an initial analysis of infrastructure requirements for DCCs aiming to support Industry 4.0, the internet of things (IoT) and the digital transformation of metrology.	D1.1
Oppermann et al. [35]	2020	To discuss the digital transformation of sovereign processes as a driving force to streamline and innovate processes for measuring instruments under legal control. It focuses on two main procedures and outlines their progress in the digital transformation, such as verification application as well as software update, and puts the related work into perspective with the AnGeWaNt—Arbeit an Geeichten Waagen fur hybride Wiegeleistungen an Nutzfahrzeugen—project [14].	D1.5
Hall and White [36]	2021	To discuss the basic requirements for uncertainty of measurement along a traceability chain and explain that standard uncertainties are needed, rather than expanded uncertainties. It explains that reporting components of uncertainty along a chain of measurements can significantly improve the quality of final results. By going beyond current practice, digital systems could become more compatible with the GUM [55].	D1.4 and D1.2
Hall and Kuster [37]	2021	To propose a layer of metrological information that enables familiar unit formats to be rendered to users as metrological support for quantities and units in digital systems. The layer should handle three independent data aspects: (i) the quantity; (ii) the measurement scale, scale type and conversion functions; and (iii) the semantics of numerical values.	D1.3
Hall and Koo [38]	2021	To present a future scenario in which digital reporting of measurement results is ubiquitous, and DCCs contain information about all components of uncertainty in a measurement result. The task of linking international measurement comparisons is used as a case study to look at the benefits of digital representation of measurement uncertainty.	D1.4
Oppermann et al. [39]	2021	To propose an approach to a distributed architecture for consolidating metrological services and data for digital transformation in legal metrology area.	D1.5
Keidel and Eichstädt [40]	2021	To demonstrate how individual processing steps can be combined into a harmonious overall digital process through the suitable selection of an IT infrastructure and knowledge of the necessary data formats. The authors used the example of the digital transformation of metrological services at PTB and provided evidence of how this approach can be translated to interoperable DQI.	D1.1 and D1.5
Boschung et al. [41]	2021	To propose an approach for DCCs based on a PDF/A-3 solution that could be a stepping stone toward the digitalization of metrological services. It presents multiple applications of this approach by fulfilling the discussed minimum requirements and satisfying the needs of both customers and laboratories.	D1.1
Softic et al. [42]	2021	To discuss a blockchain-based metrological traceability approach and evaluate how blockchain can improve data integrity and prevent data alteration in calibration certificates.	D1.2
Chalk et al. [43]	2021	To summarize and discuss the activities within the scope of the Task Group on the “Digital SI” [10], supported by the International Committee for Weights and Measures (CIPM).	D1.3
Garg et al. [44]	2021	To analyze and discuss the various aspects of the digital transformation in metrology that shall be pivotal in establishing a metrology cloud and a national DQI, thus strengthening the three main pillars: metrology, accreditation and standardization. Focusing on the Indian context, the study presents the SWOT analysis of the digital transformation in metrology previewed for the next few years in this country.	D1.5
Grasso Toro and Lehmann [45]	2021	To summarize the status of digitalization of metrology in Europe, clarifying terms and describing the digitalization strategies used by influential NMIs in some European countries. Discussion regarding data-centered and service-centered strategies aims to support a global solution that might allow every NMI to define their digitalization strategies.	D1.5
Melo et al. [46]	2021	To present the main concepts that bind legal metrology to blockchain-based technologies.	D1.5
Gadelrab et al. [17]	2021	To review a thorough analysis of the calibration process and certificate as well as the previous initiatives on DCCs.	D1.1
Smith et al. [47]	2022	To discuss the storage of uncertainty information within DCCs obtained using a Monte Carlo method. While the GUMS1 approach to uncertainty evaluation is well established [52], DCCs are a much more recent development, and the potential overlap between GUMS1 and DCCs has to be discussed in depth. The authors present two examples to demonstrate how DCCs can allow the complete set of results of a Monte Carlo calculation to be reported.	D1.3
Mustapää et al. [48]	2022	To present a method for using digital formats for metrological data, including digital signatures and distributed ledger technology (DLT), alongside DCCs and the D-SI to ensure integrity, authenticity and non-repudiation of measurement data and DCCs. The presented system was tested and validated to provide security against data cyber attacks.	D1.1 and D1.3
Oppermann et al. [49]	2022	To describe a metrological quality infrastructure in Germany that considers the strict legal framework and offers transparency, security and resilience, providing a digital transformed conformity process to the service hub and avoiding expensive re-implementation. Two exemplary use cases are described, and their benefits to the metrological service ecosystem are highlighted.	D1.5
Brown et al. [50]	2022	To discuss the recent revision of the SI [10,43] and the need to provide a similar step change in end users’ experience of the benefits that digitalization should bring. The authors argue that a digital framework for the SI is required allowing machine-readable measurement reporting, agreed metadata standards, understanding measurement uncertainty in complex systems and validation of data analysis algorithms.	D1.3
Milicevic et al. [51]	2022	To define a trust model concept for IoT blockchain, starting from the IoT device level, for analyzing the possibility of implementing a WELMEC standard [58] using the blockchain and go through levels of metrology hierarchy up to the sole definition of the measurement unit.	D1.2

Note: D1.1—Digital calibration certificates; D1.2—Digital representation of metrological traceability; D1.3—Digital representation of quantities and units of measurement; D1.4—Digital representation of measurement error and uncertainty; D1.5—Digitalization in legal metrology and quality infrastructure.

**Table 5:** Documents reviewed in the “Metrology in the Analysis of Large Amounts of Data” category.

Document	Year	Objective	Domain(s)
Emmer et al. [52]	2017	To provide an introduction thereto and describes the current challenges faced by the industry to develop a specification for 3D measurement data management.	D2.1
Majstorovic et al. [53]	2017	To define the CPM^3^ model and its structure, to develop a model knowledge base for this model and to establish total hardware and software configurations.	D2.3
Emmer et al. [54]	2017	To introduce a novel approach for a comprehensive measurement data management (MDM) that fulfills the technological requirements of Industry 4.0 for complex process chains.	D2.1
Majstorovic et al. [55]	2018	To present recent results of research on cyber–physical manufacturing metrological model—a big data analytics issue.	D2.3
D’Emilia and Gaspari [56]	2018	To describe a methodology aiming at introducing validation actions in all steps of the measurement process in a “big data” scenario, typical of an Industry 4.0 application.	D2.1
Anwer et al. [57]	2018	To address the classification problem of partitioning operations to provide a science-based solution for the development of ISO GPS partitioning standards.	D2.3
Rao et al. [58]	2018	To present the development of a 3D scanner based on digital fringe projection and to evaluate its accuracy using calibration artifact.	D2.3
Berry and Barari [59]	2019	To outline the “work-piece memory” concept and its corresponding cyber–physical system in an integrated inspection system in a high-level manner.	D2.3
Papananias et al. [60]	2019	To present an intelligent metrology informatics system to extract useful information from multistage manufacturing process data and to predict part quality characteristics using neural networks.	D2.3
Gohari et al. [61]	2019	To present a virtual replica that works in parallel with an integrated inspection system (IIS) for inspection of freeform and complex surfaces based on a metric of their geometric complexity.	D2.3
Papananias et al. [62]	2019	To discuss multistage manufacturing processes (MMPs) and to develop a probabilistic model based on Bayesian linear regression to estimate the results of final inspection associated with comparative coordinate measurement given the in-process measured coordinates.	D2.3
Gao et al. [63]	2019	To present the state-of-the-art on-machine and in-process measurement systems and sensor technologies.	D2.1 and D2.2
Majstorovic et al. [64]	2019	To present recent results of research on the building of an industrial internet of things for a cyber–physical manufacturing metrological model, in the application layer.	D2.2 and D2.3
Sabbagh et al. [65]	2020	To present recent research on enabling a cloud-based model for big data analytics within the cyber–physical manufacturing metrology model.	D2.3
Moyne et al. [66]	2020	To propose a baseline framework for digital twins (DT) technology that leverages the knowledge gained from the development of existing DT solutions and incorporates the requirements placed on DT technology by SM trends and the ultimate DT vision.	D2.3
Nasir and Sassani [21]	2021	To review the opportunities and challenges of DL for intelligent machining and tool monitoring.	D2.3
Sabbagh et al. [67]	2021	To propose a novel data curation concept that enables data mining and analytics within the recently described cyber–physical manufacturing metrology model (CPM^3^) based on organizing the metrology data into tree-based database structures using distance-based unsupervised clustering of the raw metrology data.	D2.3
Jia et al. [68]	2021	To propose a rapid and flexible calibration method based on the highly precise three-dimensional coordinate control network established by a calibration bar with an accuracy of 0.005 mm, including both estimation and optimization algorithms.	D2.4
McGregor et al. [69]	2022	To present an automated method for batch X-ray computed tomography (CT) metrology using open-source software and to investigate 48 nozzle parts produced using 11 polymer materials and three additive manufacturing (AM) processes.	D2.3
Stepanek et al. [70]	2022	To deal with the implementation of Industry 4.0 elements with a focus on metrology to ensure long-term production accuracy of CNC machine tools.	D2.3
Gallala et al. [71]	2022	To propose a digital twin (DT) approach for human–robot interactions (HRIs) in hybrid teams within the context of Industry 4.0 and smart factories.	D2.3
Tnani et al. [72]	2022	To present an efficient two-stage feature-learning approach for anomaly detection in machine processes, based on a prototype few-shot learning technique that requires a limited number of labeled samples.	D2.3
Choi et al. [73]	2022	To present a DT architecture based on an interoperable data model. It explains how to build a digital twin for the integrated control monitoring using edge devices, data analytics and realistic 3D visualization.	D2.3

Note: D2.1—Metrological analytical methods for data handling, storage, security and reliability; D2.2—Metrological traceability in IoT; D2.3—Use of cyber–physical systems, cloud computing, digital twins, artificial intelligence and machine learning in digital metrology; D2.4—Digital sensor network.

**Table 6:** Documents reviewed in the “Metrology of the Communication Systems for Digitalization” category.

Document	Year	Objective	Domain(s)
Forsstrom et al. [74]	2018	To discuss the challenges of securing the IIoT value chain.	D3.2
Thiel and Wetzlich [75]	2019	To analyze how recent regulations within the General Data Protection Regulation (2016/679/EU) and the regulation on a framework for the free flow of non-personal data in the European Union (Regulation EU 2018/1807) may be integrated into the European Metrology Cloud initiative to, e.g., guarantee that its underlying blockchain approach complies with these norms and exploits their benefits.	D3.1, D3.2, D3.3, and D3.4
Melo et al. [76]	2019	To discuss how blockchain-based technologies can improve measuring applications, evaluating two main aspects: (i) distributed measuring (DM) and (ii) decentralized surveillance.	D3.3
Peters et al. [77]	2020	To propose a security framework for measuring instruments, combining homomorphic encryption (HE) with blockchain and confidential checking of software functionality and distributed measuring instruments.	D3.2
Ačko et al. [78]	2020	To describe a formal framework for the transmission of metrology data based on the SI within the scope of the European project EMPIR 17IND02 SmartCom agreed between the European Commission and the European Association of National Metrology Institutes (Euramet). The SmartCom project aims to provide the methodological and technical foundation for the unambiguous, universal, safe and uniform communication of smart metrological data in the IoT and Industry 4.0.	D3.1, D3.2, and D3.4
Paciello et al. [79]	2020	To propose a universal metadata model for metrological complex quantities.	D3.1 and D3.2
Melo et al. [80]	2020	To propose a public key infrastructure (PKI) based on a blockchain solution, describing how to tailor this solution to deal with specific aspects related to smart meter protection.	D3.3
Nummiluikki et al. [81]	2021	To propose a platform to handle the delivery of DCCs and the connection between calibration certificate issuers and users. It introduces the features required to implement DCCs and discusses how the platform could benefit different parties in the calibration ecosystem. Additionally, the changes required for implementing the DCC platform and who should maintain the platform are discussed.	D3.2
Xiong et al. [82]	2021	To propose a digital framework for metrological information, which provides an option for NMIs to implement digital transformation.	D3.3
Moni et al. [83]	2021	To present a functional architecture to integrate NMIs in a collaborative blockchain network and to discuss the main aspects and features that an inter-NMI blockchain network must deliver.	D3.3
Sousa et al. [84]	2022	To propose a quality-assurance-focused information model named “Common Interface for Metrology Device Integration” (CIMetroI) addressing the integration of measuring devices in an IoT architecture using open standards. It provides a framework based on IEC 62264 for quality operations management (QOM) and ISO 23952:2020—Quality Information Framework (QIF) to describe the activities of quality assurance and quality control.	D3.2 and D3.4
Chen et al. [85]	2022	To develop a set of IoT controllers with integrated wireless transmission and programable logic control functions, exploring five features for programable logic controller (PLC) applications to enable users to control PLCs more easily.	D3.2

Note: D3.1—Data-based metrological infrastructure in complex industrial scenarios; D3.2—Integration of IoT, cyber–physical systems and cloud computing for efficient communication in complex industrial scenarios; D3.3—national digital quality infrastructure; D3.4—Traceability, conformity assessment and standardization.

**Table 7:** Documents reviewed in the “Metrology for Simulations and Virtual Measuring Instruments” category.

Document	Year	Objective	Domain(s)
Tieng et al. [86]	2017	To describe how the automatic virtual metrology (AVM), a system developed by the authors, coupled with target value adjustment (TVA), can be applied to facilitate the migration from mass production toward mass customization (MC), encompassing large scale, low cost, short lead time and high quality.	D4.1
Tieng et al. [87]	2018	To apply the automatic virtual metrology (AVM), a system developed by the authors, coupled with deformation fusion (DF), to deal with component deformation problems in the manufacturing of complex aerospace components, which suffer from long metrology delays and severe deformations.	D4.1
Maggipinto et al. [88]	2018	To present a deep learning (DL) method for semi-supervised feature extraction. Multi-dimensional measures used in virtual metrology (VM), such as in semiconductor manufacturing, need features to be extracted from raw data, often by hand and with specific domain knowledge, being difficult to scale and prone to information loss. The method presented herein overcomes these difficulties.	D4.1. and D4.2
Maggipinto et al. [89]	2018	To present deep learning (DL) methods for automatic feature extraction in the same manufacturing domain and a similar application of virtual metrology (VM) being presented in the previous paper [88].	D4.1 and D4.2
Maggipinto et al. [90]	2019	To present a deep learning (DL) approach for virtual metrology (VM), aiming at the semi-automatic feature extraction from two-dimensional data in the semiconductor manufacturing domain.	D4.1 and D4.2
Ferraguti et al. [91]	2019	To propose a novel method for quality assessment of polished surfaces based on augmented reality (AR) to support the operators. The surface metrology data obtained by a measurement system are directly projected on the polished component surface through an AR headset worn by the operators. The end user and the operator can directly see on the component if the quality satisfies the specifications or if some parts of the surface require further refinements.	D4.3
Hou et al. [92]	2019	To present a system for automatic visual inspection of micro-defects on semiconductor laser chip surfaces, based on virtual metrology (VM) resources and deep learning (DL) tools to handle the large volume of data that need to be classified.	D4.1 and D4.2
Hsieh et al. [93]	2019	To propose a production data traceback (PDT) mechanism that provides a virtual label to each unit produced in a continuous production process for carbon fiber components. It is an application of automatic virtual metrology (AVM) aimed at real-time total quality inspection in continuous production processes and also adopts the Advanced Manufacturing Cloud of Things (AMCoT) platform to fulfill the goal of zero defects in carbon fiber manufacturing.	D4.1
Dreyfus et al. [19]	2021	To present a SLR and an integrative conceptual framework addressing the discussion of virtual metrology (VM) use for product quality estimation in smart manufacturing. The VM framework comprises: preprocessing, quality estimation, drift detection (DD), a sample decision system (SDS), the updatability feature, the adaptability feature, a multistage architecture, machine control and a fab-wide architecture.	D4.1
Ho et al. [23]	2022	To present a SLR on augmented reality (AR) systems and their applications in the smart manufacturing context, covering the period from 2010 to 2021. There is a tendency toward interest in developing and implementing AR-assisted quality applications, currently considering three main categories: AR-based apps as a virtual lean tool, AR-assisted metrology and AR-based solutions for in-line quality control.	D4.3
Chien et al. [94]	2022	To develop a decision-based virtual metrology (VM) framework that integrates clustering and regression models to enhance the prediction and ensure the decision quality in semiconductor manufacturing processes. A number of VM models have been proposed to predict the quality characteristics of the products, but little research has been conducted to address the interrelations between the VM model and the associated decisions for advanced process control and yield enhancement.	D4.1

Note: Virtual metrology (D4.1); Deep learning and digital transformation of metrology (D4.2); Augmented reality and digital transformation of metrology (D4.3).

## Data Availability

Not applicable.

## Appendix A. Search Histories in the Web of Science and Scopus Databases

**Table A1:** Search strategy in the Web of Science database.

Ref.	Keyword Search	Documents
#1	TS = metrolog *	43,247
#2	TS = (“smart manufacturing” OR “digital manufacturing” OR “industry 4.0”)	16,200
#3	TS = (“digital calibration certificate *”)	11
#4	#1 AND #2	148
#5	#3 OR #4	156
#6	#5 AND (LIMIT-TO (PUBYEAR, 2022) OR LIMIT-TO (PUBYEAR, 2021) OR LIMIT-TO (PUBYEAR, 2020) OR LIMIT-TO (PUBYEAR, 2019) OR LIMIT-TO (PUBYEAR, 2018) OR LIMIT-TO (PUBYEAR, 2017) OR LIMIT-TO (PUBYEAR, 2016)	150
#7	#6 AND (LIMIT-TO (LANGUAGE, “English”)	144

Note: Search strategy and assessment on 4 August 2022.

**Table A2:** Search strategy in the Scopus database.

Ref.	Keyword Search	Documents
#1	TITLE-ABS-KEY (*metrolog*)	60,662
#2	TITLE-ABS-KEY (“smart manufacturing” OR “digital manufacturing” OR “industry 4.0”)	26,612
#3	TITLE-ABS-KEY(“digital calibration certificate *”)	24
#4	#1 AND #2	232
#5	#3 OR #4	250
#6	#5 AND (LIMIT-TO (PUBYEAR, 2022) OR LIMIT-TO (PUBYEAR, 2021) OR LIMIT-TO (PUBYEAR, 2020) OR LIMIT-TO (PUBYEAR, 2019) OR LIMIT-TO (PUBYEAR, 2018) OR LIMIT-TO (PUBYEAR, 2017) OR LIMIT-TO (PUBYEAR, 2016)	242
#7	#6 AND (LIMIT-TO (LANGUAGE, “English”)	238

Note: Search strategy and assessment on 4 August 2022.

**Table A3:** Document selection for the systematic literature review.

Ref.	Document Selection Step	Documents
#1	Step 1: Retrieved documents after removing duplicates	247
#2	Step 3: Panel assessment of documents by potential relevance to this SLR: scores of 3 and 2	160
#3	Step 4: Selected documents published in 2016–2020: number of citations ≥ 5	37
#4	Step 4: Selected documents published in 2021–2022 regardless of the number of citations	33
#5	Selected articles for SLR	70
